# Supplementary material for: Constructing molecular bridge for high-efficiency and stable perovskite solar cells based on P3HT
Source: Nat Commun. 2022 Nov 17;13:7020. doi: 10.1038/s41467-022-34768-7 (PMC9672063; doi:10.1038/s41467-022-34768-7)
Supplement: Supplementary file 1 — Supplementary Information [file 41467_2022_34768_MOESM1_ESM.pdf]

Supporting Information

Constructing Molecular Bridge for High-Efficiency and Stable Perovskite Solar Cells based on P3HT

Dongdong Xu,[1] Zhiming Gong, [1] Yue Jiang, [1*] Yancong Feng, [2*] Zhen Wang, [1] Xingsen Gao, [1] Xubing Lu, [1] Guofu Zhou, [2] Jun-Ming Liu, [3] Jinwei Gao [1*]

1. Institute for Advanced Materials and Guangdong Provincial Key Laboratory of Optical Information Materials and Technology, South China Academy of Advanced Optoelectronics, South China Normal University, Guangzhou 510006, China.
2. Institute of Electronic Paper Displays, South China Academy of Advanced Optoelectronics, South China Normal University, Guangzhou 510006, China.
3. Laboratory of Solid-State Microstructures, Nanjing University, Nanjing 210093, China.

E-mail: yuejiang@m.scnu.edu.cn (Y.J.), fengyancong@m.scnu.edu.cn (Y.F.), gaojinwei@m.scnu.edu.cn (J.G.)

Supplementary Methods

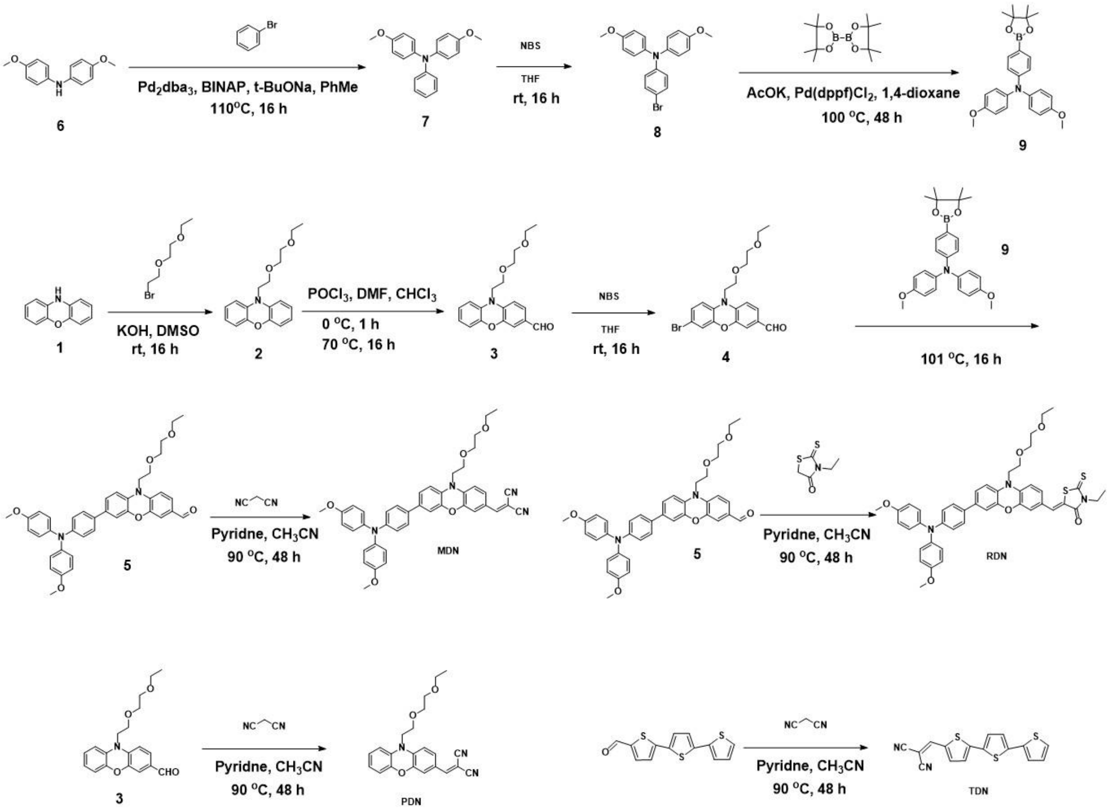

Supplementary Fig. 1. Synthetic routes of MDN, RDN, PDN, and TDN.

10-(2-(2-ethoxyethoxy)ethyl)-10H-phenoxazine (2):

The KOH (1.36 g, 24 mmol) was added slowly to a solution of 10H-phenoxazine (2.2 g, 12 mmol) and 1-bromo-2-(2-ethoxyethoxy)ethane (2.4 g, 12 mmol) in 30 mL tetrahydrofuran at room temperature, then the mixture was stirred for 16 hours at room temperature. The mixture was diluted with dichloromethane and washed with water. The organic layer was dried over Na_2SO_4 and evaporated under a vacuum. The crude product was purified by column chromatography (SiO_2, petroleum ether 40−60 °C / ethyl acetate = 20/1 v/v) and to obtain compound **2** (3.1 g, 86.4 % yield) as a red oil.

^{1}H NMR (600 MHz, DMSO-*d*6): 6.83−6.80 (m, 2H), 6.75−6.74 (m, 2H), 6.67−6.63 (m, 4H), 3.76 (t, *J* = 6.0 Hz, 2H), 3.65 (t, *J* = 6.0 Hz, 2H), 3.57−3.55 (m, 2H), 3.47−3.46 (m, 2H), 3.42−3.39 (m, 2H), 1.07 (t, *J* = 7.2 Hz, 2H);

^{13}C NMR (150 MHz, DMSO-*d*6):144.54, 133.58, 124.37, 121.43, 115.41, 113.00, 70.66, 69.73, 67.13, 66.10, 44.46, 15.57;

FTMS (APCI) calcd for $C_{18}H_{22}NO_3$ ($M+H^+$): 300.1600; found: 300.1591.

10-(2-(2-ethoxyethoxy)ethyl)-10H-phenoxazine-3-carbaldehyde (3):

The $POCl_3$ (3.07 g, 20.1 mmol) was added slowly to a solution of compound **2** (2 g, 6.7 mmol) and anhydrous N,N-dimethylformamide (4 mL) in 20 mL anhydrous chloroform at 0~5 °C under argon atmosphere, the mixture was stirred for 1 hour at 0~5 °C, and then the mixture was stirred for 16 hours at 65 °C. After the reaction completed, the mixture was quenched with sodium hydroxide solution. Then the mixture was extracted with dichloromethane for three times, and then the combined organic phase was dried over Na_2SO_4 and evaporated under vacuum. The crude product was purified by column chromatography (SiO_2, petroleum ether 40−60 °C / ethyl acetate = 5/1 v/v) and to obtain compound **3** (2 g, 91.3 % yield) as a yellow oil.

^1H NMR (600 MHz, DMSO-*d*6): 9.65 (s, 1H), 7.39 (dd, J_1 = 8.4 Hz, J_2 = 1.8 Hz, 1H), 7.00 (d, J = 1.8 Hz, 1H), 6.92 (d, J = 8.4 Hz, 2H), 6.86−6.85 (m, 2H), 6.77−6.74 (m, 1H), 6.70−6.68 (m, 1H), 3.86 (t, J = 6.0 Hz, 2H), 3.68 (t, J = 6.0 Hz, 2H), 3.57−3.55 (m, 2H), 3.46−3.44 (m, 2H), 3.41−3.37 (m, 2H), 1.05 (t, J = 7.2 Hz, 2H);

^{13}C NMR (150 MHz, DMSO-*d*6): 190.44, 144.61, 144.21, 139.55, 131.77, 130.07, 129.02, 124.61, 123.01, 115.69, 113.97, 113.71, 112.78, 70.74, 69.71, 67.20, 66.11, 44.53, 15.56;

FTMS (APCI) calcd for C19H22NO4 ($M+H^+$): 328.1549; found: 328.1528.

7-bromo-10-(2-(2-ethoxyethoxy)ethyl)-10H-phenoxazine-3-carbaldehyde (4):

The N-Bromosuccinimide (547 mg, 3.22 mmol) in tetrahydrofuran (5 mL) was added slowly to a solution of compound **3** (960 mg, 2.94 mmol) in tetrahydrofuran (5 mL) at 0~5 °C, the mixture was stirred for 1 hours at 0~5 °C,

and then the mixture was stirred for 16 hours at room temperature. After the reaction completed, the mixture was quenched with saturated sodium bicarbonate solution. Then the mixture was extracted with dichloromethane for three times, and then the combined organic phase was dried over Na_2SO_4 and evaporated under vacuum. The crude product was used in next step without further purification.

^{1}H NMR (600 MHz, DMSO-*d*6): 9.65 (s, 1H), 7.40 (dd, J_1 = 8.4 Hz, J_2 = 1.8 Hz, 1H), 7.00−6.98 (m, 2H), 6.92 (d, *J* = 8.4 Hz, 1H), 6.86 (d, *J* = 2.4 Hz, 1H), 6.79 (d, *J* = 9.0 Hz, 1H), 3.83 (t, *J* = 6.0 Hz, 2H), 3.66 (t, *J* = 6.0 Hz, 2H), 3.55−3.54 (m, 2H), 3.45−3.43 (m, 2H), 3.39−3.36 (m, 2H), 1.04 (t, *J* = 7.2 Hz, 2H);

^{13}C NMR (150 MHz, DMSO-*d*6): 190.48, 145.07, 144.21, 138.92, 131.54, 130.26, 129.13, 127.01, 118.27, 115.60, 113.96, 113.44, 113.08, 70.76, 69.71, 67.18, 66.11, 44.69, 15.54;

FTMS (APCI) calcd for $C_{26}H_{31}BNO_2S_2$ (M+H$^+$): 464.1889; found: 464.1890.

7-(4-(bis(4-methoxyphenyl)amino)phenyl)-10-(2-(2-ethoxyethoxy)ethyl)-10H-phenoxazine-3-carbaldehyde (5):

A mixture of compound **4** (406 mg, 1.0 mmol), compound **9** (431 mg, 1.0 mmol), $Pd(PPh_3)_4$ (11 mg, 0.01 mmol), potassium carbonate (280 mg, 2.0 mmol) in 1,4-dioxane (5 mL) and water (1 mL) was stirred at 100 ^oC under argon atmosphere for 16 hours. After the mixture was cooled to room temperature, 50 mL of water were added and water phase was extracted with dichloromethane for three times, and then the combined organic phase was dried over Na_2SO_4. After the solvent was evaporated under vacuum, the crude product was purified by column chromatography (SiO_2, petroleum ether 40−60 ^oC / ethyl acetate = 5/1 v/v) to give compound **5** as a yellow solid (530 mg, 84.1 % yield).

^{1}H NMR (600 MHz, DMSO-*d*6): 9.65 (s, 1H), 7.46 (d, *J* = 9.0 Hz, 2H), 7.39 (dd, J_1 = 8.4 Hz, J_2 = 1.8 Hz, 1H), 7.09 (dd, J_1 = 8.4 Hz, J_2 = 1.8 Hz, 1H), 7.05−7.02 (m, 5H), 6.94−6.91 (m, 6H), 6.88 (d, *J* = 8.4 Hz, 1H), 6.78 (d, *J* = 9.0

Hz, 1H), 3.90−3.87 (m, 2H), 3.75 (s, 6H),3.70 (t, J = 6.0 Hz, 2H), 3.57−3.56 (m, 2H), 3.46−3.45 (m, 2H), 3.41−3.37 (m, 2H), 1.05 (t, J = 7.2 Hz, 2H);

^{13}C NMR (150 MHz, DMSO-$d6$): 190.43, 156.27, 148.15, 144.54, 144.45, 140.47, 139.29, 134.74, 130.54, 130.24, 130.04, 129.08, 127.21, 126.90, 121.56, 119.92, 115.45, 114.33, 113.73, 112.82, 112.78, 70.77, 69.71, 67.26, 66.12, 55.71, 44.53, 40.53, 15.56;

FTMS (APCI) calcd for $C_{19}H_{21}BrNO_4$ (M+H$^+$): 406.0654; found: 406.0645.

4-methoxy-N-(4-methoxyphenyl)-N-phenylaniline (7):

A mixture of bis(4-methoxyphenyl)amine (2.29 g, 10 mmol), bromobenzene (1.73 g, 11 mmol), Pd_2dba_3 (91 mg, 0.1 mmol), t-Bu_3PHBF_4 (116 mg, 0.4 mmol), sodium tert-butoxide (1.44 g, 15 mmol) in anhydrous toluene (20 mL) was stirred at 110 ^oC under argon atmosphere for 16 hours. After evaporating the solvent under reduced pressure, the remaining crude product was purified by column chromatography (SiO_2, petroleum ether 40−60 ^oC /ethyl acetate = 25/1 v/v) to give compound **7** as a yellow oil (2.73 g, 89.5% yield).

^{1}H NMR (600 MHz, $CDCl_3$): 7.19 (t, J = 7.2 Hz, 2H), 7.08−7.05 (m, 4H), 6.96 (d, J = 7.8 Hz, 2H) , 6.89 (t, J = 7.2 Hz, 1H), 6.86−6.84 (m, 4H), 3.82 (s, 6H).

4-bromo-N,N-bis(4-methoxyphenyl)aniline (8):

The N-Bromosuccinimide (1.59 g, 8.95 mmol) in tetrahydrofuran (15 mL) was added slowly to a solution of compound **7** (2.73 mg, 8.95 mmol) in tetrahydrofuran (15 mL) at 0~5 ^oC, the mixture was stirred for 1 hours at 0~5 ^oC, and then the mixture was stirred for 16 hours at room temperature. After the reaction completed, the mixture was quenched with saturated sodium bicarbonate solution. Then the mixture was extracted with dichloromethane for three times, and then the combined organic phase was dried over Na_2SO_4 and evaporated under vacuum. The crude product was used in next step without further purification.

4-methoxy-N-(4-methoxyphenyl)-N-(4-(4,4,5,5-tetramethyl-1,3,2-dioxaborolan-2-yl)phenyl)aniline (9):

A mixture of compound **8** (3.43 g, 8.95 mmol), bis(pinacolato)diboron (2.72 g, 10.74 mmol), Pd(dppf)Cl_2 (66 mg, 0.09 mmol), Potassium Acetate (1.8 g, 17.9 mmol) in anhydrous 1,4-dioxane (30 mL) was stirred at 100 °C under argon atmosphere for 72 hours. After evaporating the solvent under reduced pressure, the remaining crude product was purified by column chromatography (SiO_2, petroleum ether 40−60 °C /ethyl acetate = 25/1 v/v) to give compound **9** as a yellow solid (3.63 g, 94 % yield).

^{1}H NMR (600 MHz, $CDCl_3$): 7.62 (t, J = 9.0 Hz, 2H), 7.10−7.07 (m, 4H), 6.89 (d, J = 9.0 Hz, 2H) , 6.87−6.83 (m, 4H), 3.82 (s, 6H), 1.34 (s, 12H).

2-((7-(4-(bis(4-methoxyphenyl)amino)phenyl)-10-(2-(2-ethoxyethoxy)ethyl)-10H-phenoxazin-3-yl)methylene)malononitrile (MDN):

A mixture of compound **5** (520 mg, 0.83 mmol), malononitrile (110 mg, 1.66 mmol), pyridine (200 g, 2.46 mmol) in acetonitrile (10 mL) was stirred at 90 °C under argon atmosphere for 72 hours. After evaporating the solvent under reduced pressure, the remaining crude product was purified by column chromatography (SiO_2, petroleum ether 40−60 °C /ethyl acetate = 2/1 v/v) to give **MDN** as a red solid (510 mg, 90.6 % yield) (Tg = 64 °C, Td = 392 °C).

^{1}H NMR (600 MHz, DMSO-d6): 9.65 (s, 1H), 7.46 (d, J = 9.0 Hz, 2H), 7.39 (dd, J_1 = 8.4 Hz, J_2 = 1.8 Hz, 1H), 7.09 (dd, J_1 = 8.4 Hz, J_2 = 1.8 Hz, 1H), 7.05−7.02 (m, 5H), 6.94−6.91 (m, 6H), 6.88 (d, J = 8.4 Hz, 1H), 6.78 (d, J = 9.0 Hz, 1H), 3.90−3.87 (m, 2H), 3.75 (s, 6H),3.70 (t, J = 6.0 Hz, 2H), 3.57−3.56 (m, 2H), 3.46−3.45 (m, 2H), 3.41−3.37 (m, 2H), 1.05 (t, J = 7.2 Hz, 3H);

^{13}C NMR (150 MHz, DMSO-d6): 190.43, 156.27, 148.15, 144.54, 144.45, 140.47, 139.29, 134.74, 130.54, 130.24, 130.04, 129.08, 127.21, 126.90, 121.56, 119.92, 115.45, 114.33, 113.73, 112.82, 112.78, 70.77, 69.71, 67.26, 66.12, 55.71, 44.53, 40.53, 15.56;

FTMS (APCI) calcd for $C_{42}H_{39}N_4O_5$ ($M+H^+$): 679.2920; found: 679.2924.

(Z)-5-((7-(4-(bis(4-methoxyphenyl)amino)phenyl)-10-(2-(2-ethoxyethoxy)ethyl)-10H-phenoxazin-3-yl)methylene)-3-ethyl-2-thioxothiazolidin-4-one (RDN):

A mixture of compound **5** (518 mg, 0.82 mmol), 3-ethylrhodanine (264 mg, 1.64 mmol), pyridine (200 g, 2.46 mmol) in acetonitrile (10 mL) was stirred at 90 °C under argon atmosphere for 48 hours. After evaporating the solvent under reduced pressure, the remaining crude product was purified by column chromatography (SiO_2, petroleum ether 40−60 °C /ethyl acetate = 2/1 v/v) to give **RDN** as a red solid (400 mg, 63.1% yield) (*T*g = 68 °C, *T*d = 382 °C).

^{1}H NMR (600 MHz, DMSO-*d*6): 7.56 (s, 1H), 7.41 (d, *J* = 8.4 Hz, 2H), 7.11−7.02 (m, 7H), 6.93−6.88 (m, 7H), 6.76−6.83 (m, 4H), 4.04−4.01 (m, 2H), 3.89−3.84 (m, 2H), 3.75 (s, 6H), 3.70−3.67 (m, 2H), 3.57−3.56 (m, 2H), 3.47−3.45 (m, 2H), 3.41−3.37 (m, 2H), 1.17 (t, *J* = 7.2 Hz, 3H), 1.04 (t, *J* = 7.2 Hz, 3H);

^{13}C NMR (150 MHz, DMSO-*d*6): 171.01, 156.29, 147.67, 144.87, 144.42, 140.46, 135.45, 134.89, 132.82, 131.27, 127.24, 126.86, 121.56, 119.87, 115.46, 112.81, 70.77, 69.72, 67.32, 67.25, 66.13, 55.72, 40.52, 31.43, 22.54, 15.59, 14.44, 12.40;

FTMS (APCI) calcd for $C_{44}H_{44}N_3O_6S_2$ ($M+H^+$): 774.2672; found: 774.2676.

2-((10-(2-(2-ethoxyethoxy)ethyl)-10H-phenoxazin-3-yl)methylene)malononitrile (PDN):

A mixture of compound **3** (560 mg, 1.71 mmol), malononitrile (226 mg, 3.42 mmol), pyridine (406 g, 5.14 mmol) in acetonitrile (10 mL) was stirred at 90 °C under argon atmosphere for 48 hours. After evaporating the solvent under reduced pressure, the remaining crude product was purified by column chromatography (SiO_2, petroleum ether 40−60 °C /ethyl acetate = 3/1 v/v) to give **PDN** as a blood red solid (600 mg, 93.4 % yield).

^{1}H NMR (600 MHz, $CDCl_3$-*d*): 7.36 (s, 1H), 7.27−7.26 (d, *J* = 6.0 Hz, 1H), 6.85−6.82 (t, *J* =9.0 Hz, 1H), 6.80−6.77 (t, *J* =9.0 Hz, 1H), 6.69−6.66 (m, 3H), 3.84−3.82 (t, *J* =6.0 Hz, 2H), 3.77−3.75 (t, *J* =6.0 Hz, 2H), 3.66−3.65 (m, 2H), 3.58−3.56 (m, 2H), 3.53−3.50 (m, 2H), 1.22−1.19 (t, *J* = 8.6 Hz, 3H);

^{13}C NMR (150 MHz, $CDCl_3$-*d*): 157.04, 144.76, 144.48, 140.06, 130.85, 130.64, 124.09, 139.65, 115.96, 115.07, 113.86, 112.97, 111.75, 71.20, 69.89, 67.16, 66.81, 44.85, 29.71, 15.17;

FTMS (APCI) calcd for $C_{22}H_{21}N_3O_3$ ($M+H^+$): 375.1583; found: 375.1687.

2-([2,2':5',2''-terthiophen]-5-ylmethylene)malononitrile (TDN):

A mixture of [2,2':5',2''-terthiophene]-5-carbaldehyde (276 mg, 1 mmol), malononitrile (132 mg, 2 mmol), pyridine (237 g, 3 mmol) in acetonitrile (15 mL) was stirred at 90 °C under argon atmosphere for 48 hours. After evaporating the solvent under reduced pressure, the remaining crude product was purified by column chromatography (SiO_2, petroleum ether 40−60 °C /dichloromethane = 1/1 v/v) to give **TDN** as a dark red solid (280 mg, 86.4 % yield).

^{1}H NMR (600 MHz, $CDCl_3$-*d*): 7.76 (s, 1H), 7.64−7.63 (d, *J* = 6.8 Hz, 1H), 7.36−7.35 (d, *J* = 6.0 Hz, 1H), 7.631−7.30 (d, *J* = 6.0 Hz, 1H), 7.26−7.25 (d, *J* = 6.2 Hz, 2H), 7.17−7.16 (d, *J* = 5.8 Hz, 1H), 7.07−7.06 (d, *J* =6.9 Hz, 1H);

^{13}C NMR (150 MHz, $CDCl_3$-*d*): 150.05, 149.08, 140.65, 140.19, 136.16, 133.53, 133.42, 128.25, 128.21, 125.95, 124.99, 124.95, 124.37, 114.29, 113.49, 76.04;

FTMS (APCI) calcd for $C_{42}H_{39}N_4O_5$ ($M+H^+$): 323.985; found: 323.9945.

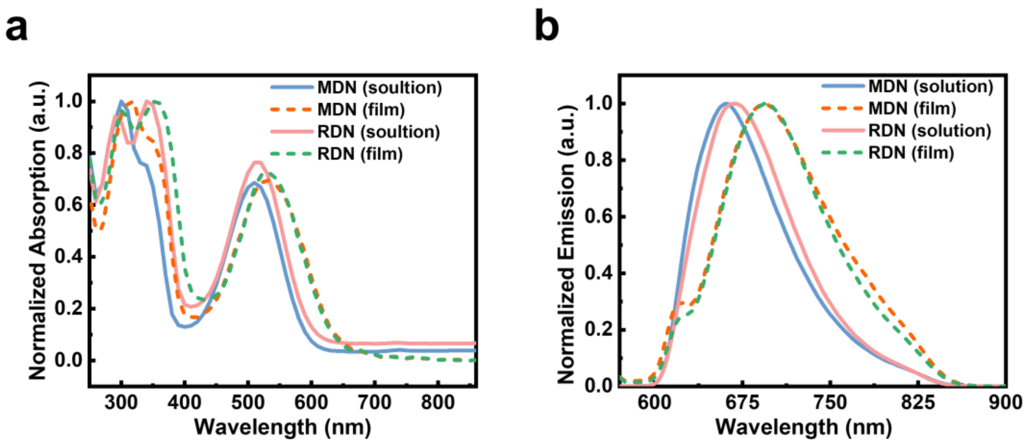

Supplementary Fig. 2. (**a**) UV–vis absorption spectra and (**b**) photoluminescence spectra of materials in THF solution and as thin films.

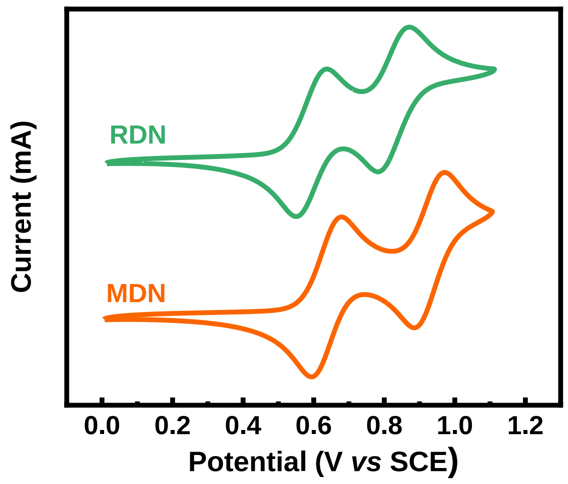

Supplementary Fig. 3. CV curves of MDN and RDN.

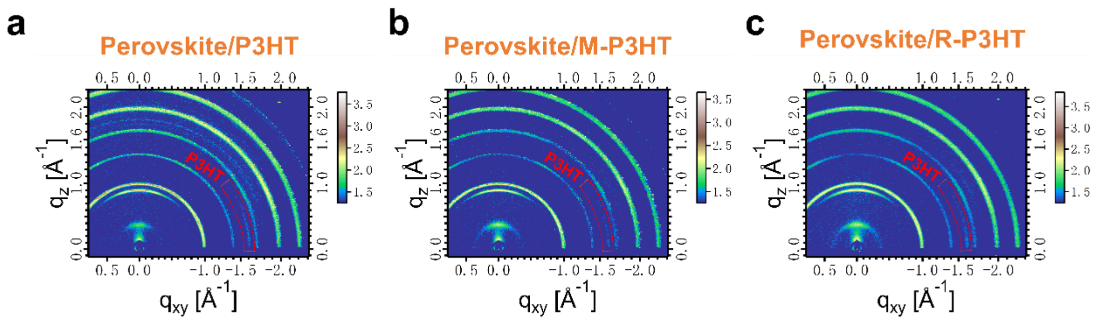

Supplementary Fig. 4. 2D GIWAXS patterns based on the structures of (**a**) Si-wafers/perovskite/P3HT, (**b**) Si-wafers/perovskite/M-P3HT, and (**c**) Si-wafers/perovskite/R-P3HT.

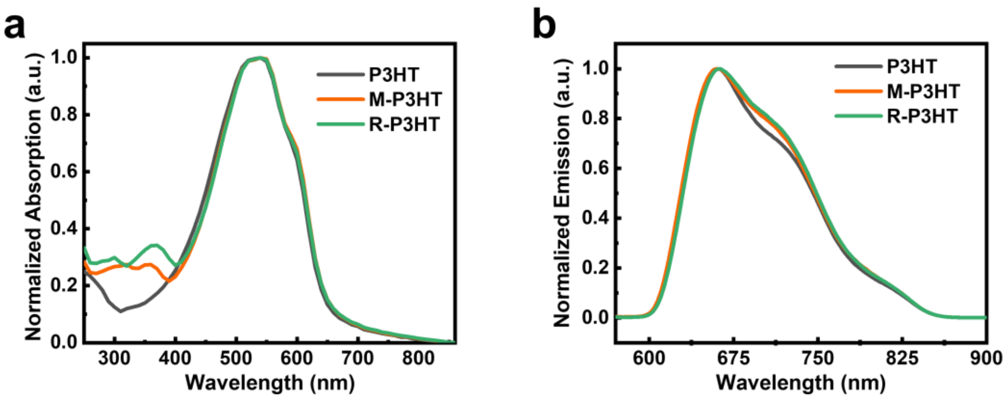

Supplementary Fig. 5. (**a**) UV–vis absorption spectra and (**b**) photoluminescence spectra of P3HT, M-P3HT, and R-P3HT films.

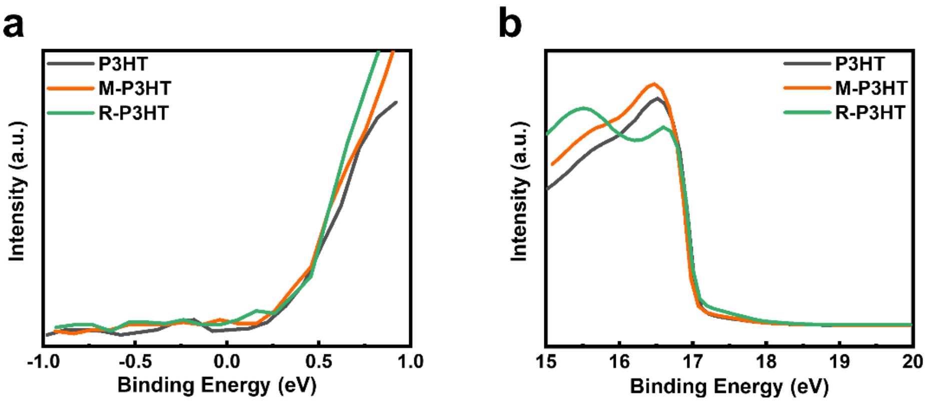

Supplementary Fig. 6. (**a**) HOMO region and (**b**) cut-off energy region of UPS.

Supplementary Table 1. Optical and electrochemical properties.

Sample	λ_{max} (nm)		λ_{em} (nm)		E_g (eV)	E_{HOMO} (eV)	E_{LUMO} (eV)
**MDN**	509^{s}	532^{f}	661^{s}	694^{f}	2.03	-5.31	-3.28
**RDN**	514^{s}	532^{f}	667^{s}	694^{f}	2.03	-5.26	-3.24
**P3HT**	538		660		1.93	-4.58	-2.65
**M-P3HT**	538		660		1.93	-4.65	-2.72
**R-P3HT**	538		661		1.93	-4.61	-2.68

^{s}Results from absorption in THF solution. ^{f}Results from absorption of films.

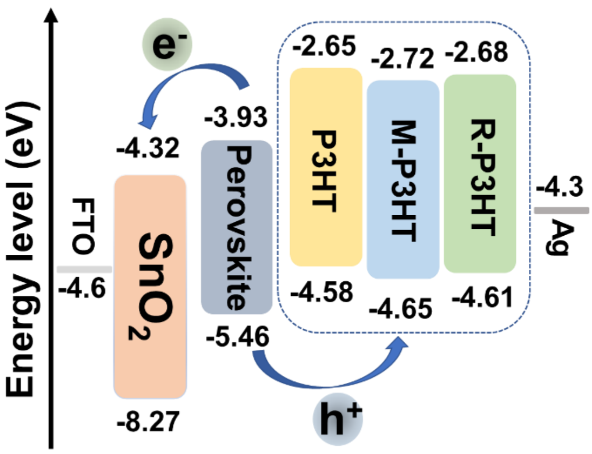

Supplementary Fig. 7. Band-edges of the stacked films in the device.

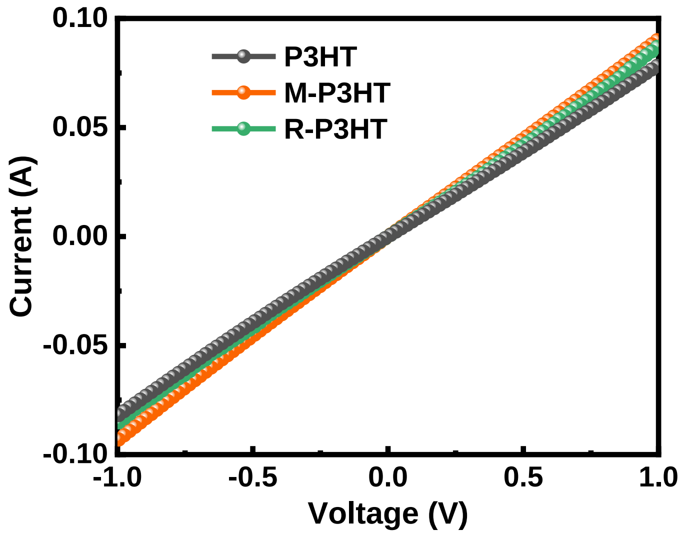

Supplementary Fig. 8. Conductivity of P3HT, M-P3HT and R-P3HT films.

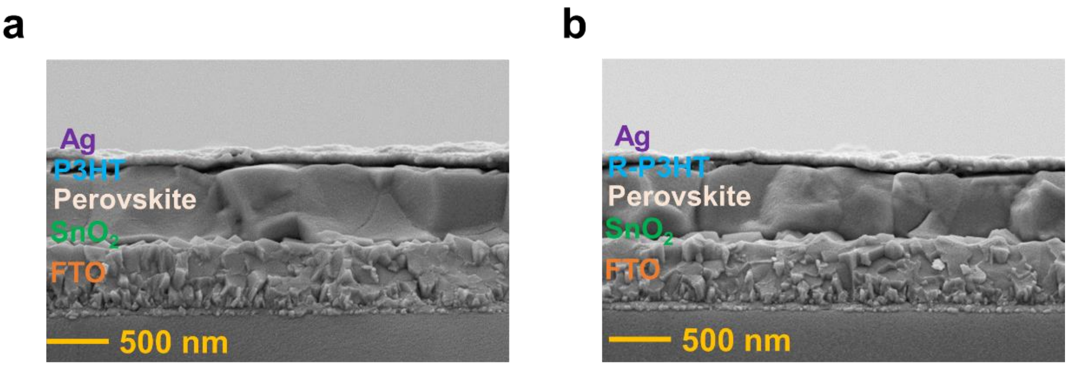

Supplementary Fig. 9. Cross-sectional SEM images of PSCs with (**a**) P3HT, and (**b**) R-P3HT.

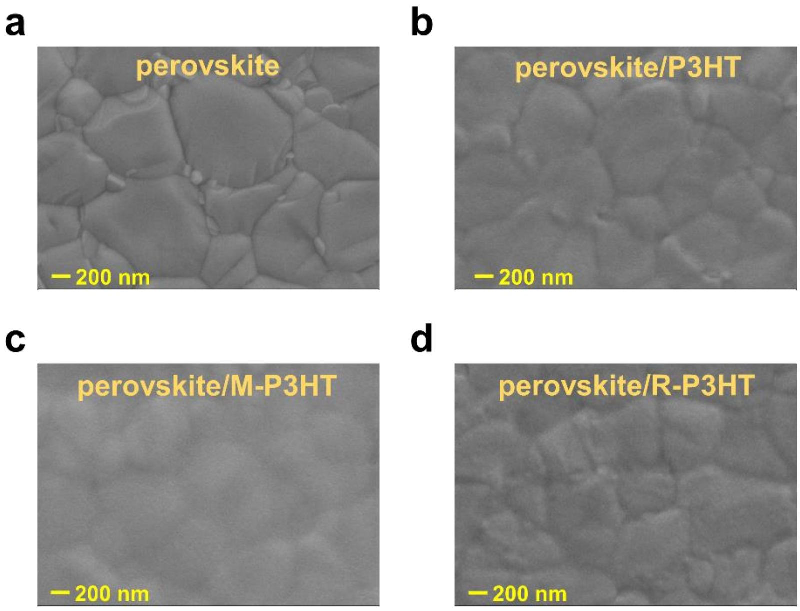

Supplementary Fig. 10. The top view SEM images with fresh devices of (**a**) perovskite, (**b**) perovskite/P3HT, (**c**) perovskite/M-P3HT, and (**d**) perovskite/R-P3HT.

Supplementary Table 2. Photovoltaic parameters of champion devices based on different HTMs measured under AM 1.5G Illumination.

Device	V_{OC} (V)	J_{SC} ($mA \cdot cm^{-2}$)	*FF* (%)	*PCE* (%)
**P3HT-REV[a)]**	0.92	23.21	58.73	12.48
**P3HT-FOR[b)]**	0.82	23.04	36.75	6.98
**M-P3HT-REV**	1.16	24.58	80.17	22.87
**M-P3HT-FOR**	1.16	24.44	78.61	22.28
**R-P3HT-REV**	1.05	23.90	66.54	16.66
**R-P3HT-FOR**	1.00	23.90	58.19	13.87

[a)] Reverse scan; [b)] Forward scan.

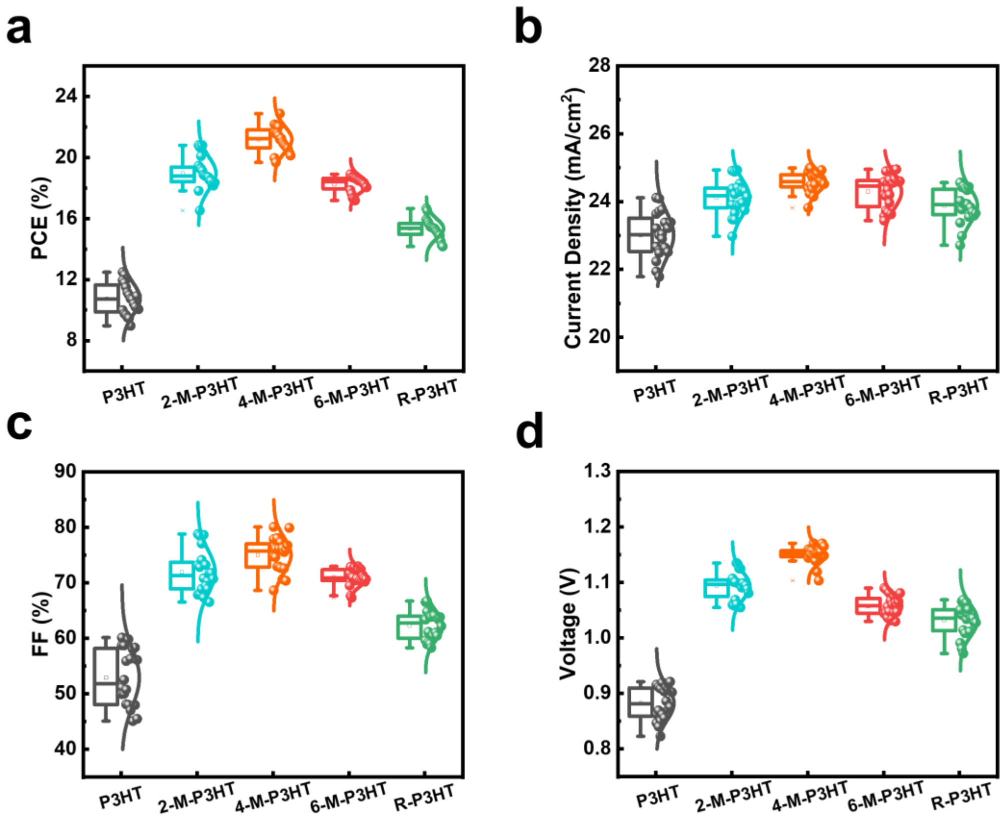

Supplementary Fig. 11. The statistical distributions of 20 devices with different concentrations of MDN and RDN of (**a**) *PCE*, (**b**) J_{SC}, (**c**) *FF*, and (**d**) V_{OC}.

Supplementary Table 3. Comparison of the device performance obtained with different HTM. The average values and standard deviations are calculated from 20 devices fabricated at the same conditions.

Device	V_{OC} (V)	J_{SC} (mA·cm^{-2})	*FF* (%)	*PCE* (%)
**P3HT**	0.88 ± 0.03	23.01 ± 0.67	52.91 ± 5.14	10.73 ± 1.03
**2-M-P3HT**	1.09 ± 0.02	24.12 ± 0.51	71.94 ± 3.85	18.96 ± 1.05
**4-M-P3HT**	1.15 ± 0.02	24.55 ± 0.32	75.02 ± 3.09	21.19 ± 0.83
**6-M-P3HT**	1.06 ± 0.02	24.29 ± 0.48	70.96 ± 1.57	18.23 ± 0.52
**R-P3HT**	1.03 ± 0.03	23.87 ± 0.49	62.30 ± 2.60	15.33 ± 0.64

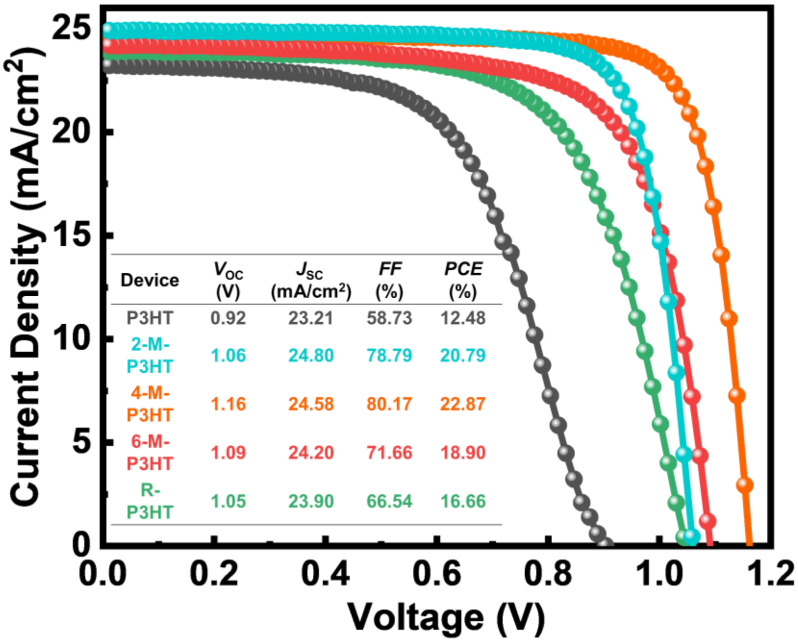

Supplementary Fig. 12. *J–V* characteristics of the best-performing based on the different HTMs (P3HT, 2-P3HT, 4-P3HT, 6-P3HT, and R-P3HT, respectively).

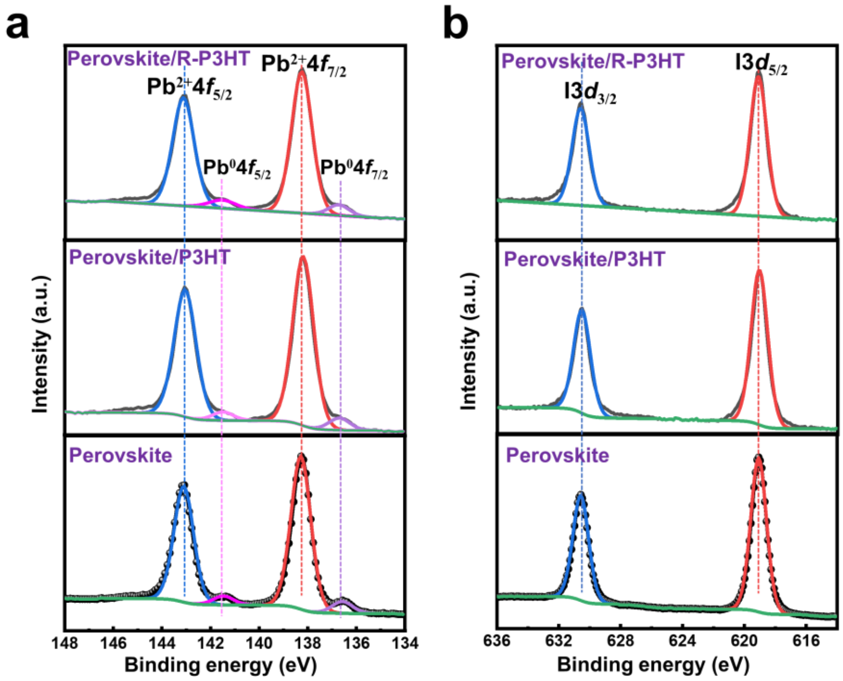

Supplementary Fig. 13. High-resolution XPS spectra of (**a**) Pb 4*f* spectra, and (**b**) I 3*d* spectra.

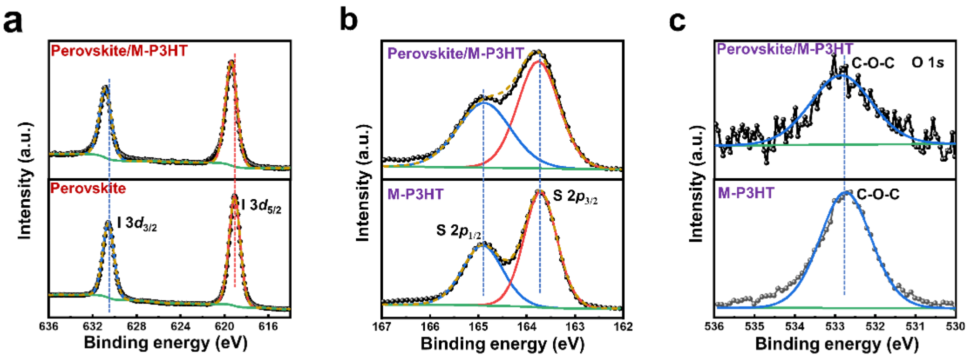

Supplementary Fig. 14. High-resolution XPS spectra of (**a**) I 3*d* spectra, (**b**) S 2*p* spectra, and (**c**) O 1*s* spectra.

Supplementary Table 4. Time-resolved PL decay fitting parameters.

Device	A_1	τ_1	A_2	τ_2	τ_{ave}
**perovskite**	2.09	32.54	0.49	1192.64	1071.71
**perovskite/P3HT**	0.47	62.28	0.11	633.75	464.75
**perovskite/R-P3HT**	0.49	29.81	0.08	525.17	397.38
**perovskite/M-P3HT**	0.65	12.62	0.05	416.51	302.38

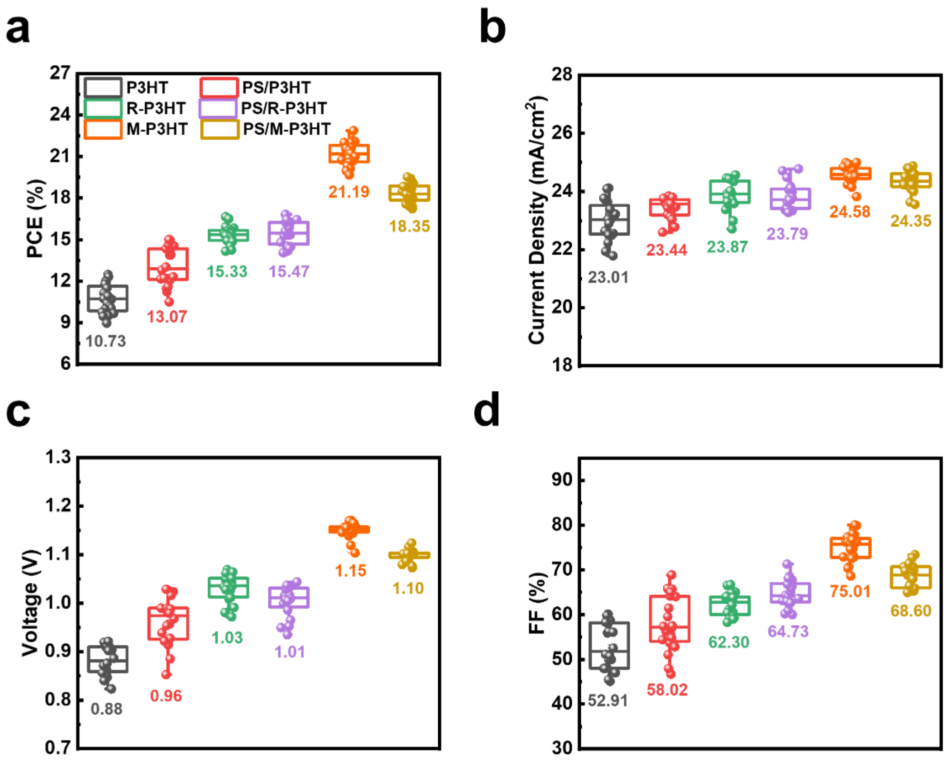

Supplementary Fig. 15. The statistical distributions of 20 devices w/ or w/o PS interface of (**a**) *PCE*, (**b**) J_{SC}, (**c**) V_{OC}, and (**d**) *FF*.

Supplementary Table 5. Comparison of the device performance obtained w/ or w/o PS. The average values and standard deviations are calculated from 20 devices fabricated at the same conditions.

Device	V_{OC} (V)	J_{SC} (mA·cm^{-2})	*FF* (%)	*PCE* (%)
**P3HT**	0.88 ± 0.03	23.01 ± 0.67	52.91 ± 5.14	10.73 ± 1.03
**PS/P3HT**	0.96 ± 0.05	23.44 ± 0.35	58.02 ± 6.19	13.07 ± 1.36
**M-P3HT**	1.15 ± 0.02	24.55 ± 0.32	75.02 ± 3.09	21.19 ± 0.83
**PS/M-P3HT**	1.10 ± 0.01	24.34 ± 0.37	68.60 ± 2.65	18.35 ± 0.69
**R-P3HT**	1.03 ± 0.03	23.87 ± 0.49	62.30 ± 2.60	15.33 ± 0.64
**PS/R-P3HT**	1.01 ± 0.03	23.79 ± 0.47	64.73 ± 2.98	15.47 ± 0.83

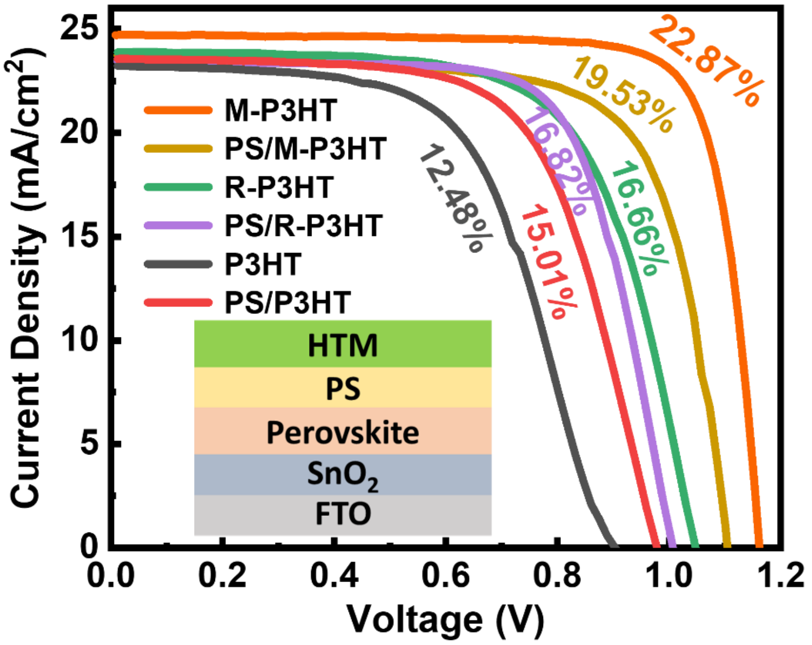

Supplementary Fig. 16. The *J–V* curves of the champion devices based on the w/ or w/o PS interface.

Supplementary Table 6. Performances of the champion devices based on the w/ or w/o PS interface.

Device	*V*oc (V)	*J*sc ($mA\cdot cm^{-2}$)	*FF* (%)	*PCE* (%)
**P3HT**	0.92	23.21	58.73	12.48
**PS/P3HT**	0.98	23.57	65.03	15.01
**M-P3HT**	1.16	24.58	80.17	22.87
**PS/M-P3HT**	1.10	24.79	71.43	19.53
**R-P3HT**	1.05	23.90	66.54	16.66
**PS/R-P3HT**	1.01	23.42	71.31	16.82

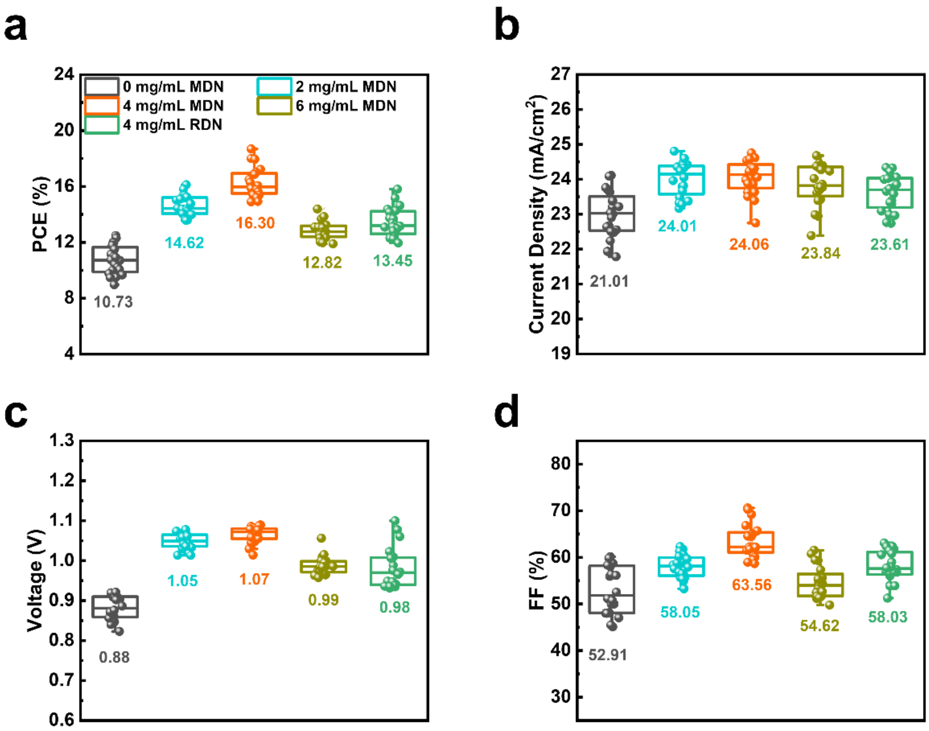

Supplementary Fig. 17. The statistical distributions of 20 devices with different concentrations of MDN and RDN interface of (**a**) *PCE*, (**b**) J_{SC}, (**c**) V_{OC}, and (**d**) *FF*.

Supplementary Table 7. Comparison of the device performance obtained with different concentrations of MDN and RDN interface. The average values and standard deviations are calculated from 20 devices fabricated at the same conditions.

Device	V_{OC} (V)	J_{SC} (mA·cm^{-2})	*FF* (%)	*PCE* (%)
**P3HT**	0.88 ± 0.03	23.01 ± 0.67	52.91 ± 5.14	10.73 ± 1.03
**2mg/ml MDN**	1.05 ± 0.02	24.01 ± 0.49	58.05 ± 2.48	14.62 ± 0.74
**4mg/ml MDN**	1.07 ± 0.02	24.06 ± 0.50	63.56 ± 3.60	16.30 ± 1.06
**6mg/ml RDN**	0.99 ± 0.02	23.84 ± 0.60	54.62 ± 3.43	12.85 ± 0.65
**4mg/ml RDN**	0.98 ± 0.05	23.61 ± 0.51	58.03 ± 3.33	13.45 ± 1.04

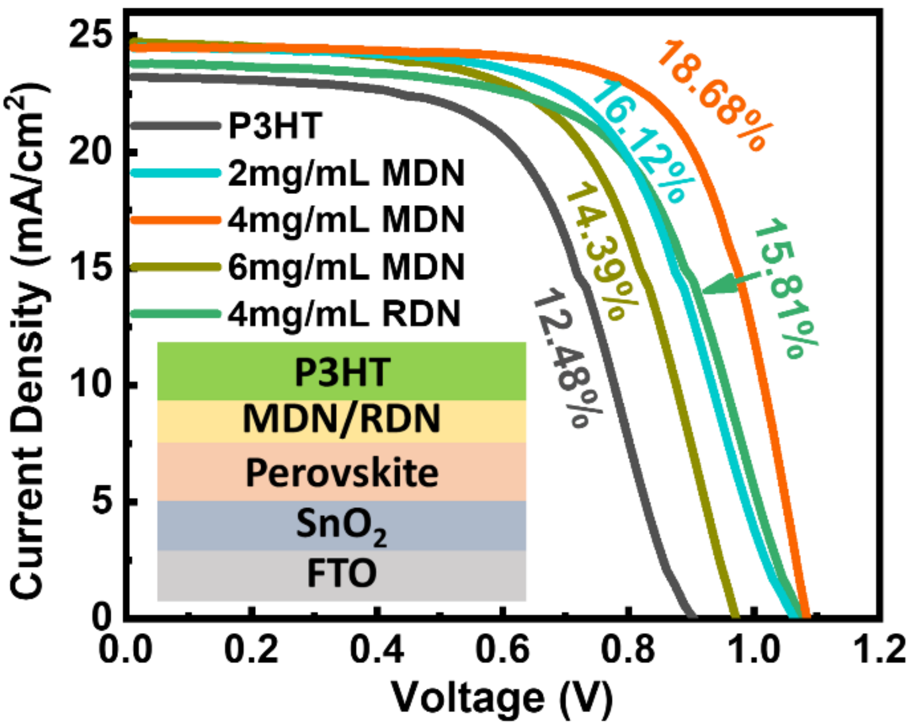

Supplementary Fig. 18. The *J–V* curves of the champion devices based on the different concentrations of MDN and RDN interface.

Supplementary Table 8. Performances of the champion devices based on the different concentration of MDN and RDN interface.

Device	V_{OC} (V)	J_{SC} ($mA \cdot cm^{-2}$)	*FF* (%)	*PCE* (%)
**P3HT**	0.92	23.21	58.73	12.48
**2mg/ml MDN**	1.07	24.46	61.74	16.12
**4mg/ml MDN**	1.09	24.49	70.27	18.68
**6mg/ml MDN**	0.97	24.02	61.51	14.39
**4mg/ml RDN**	1.08	23.77	61.71	15.81

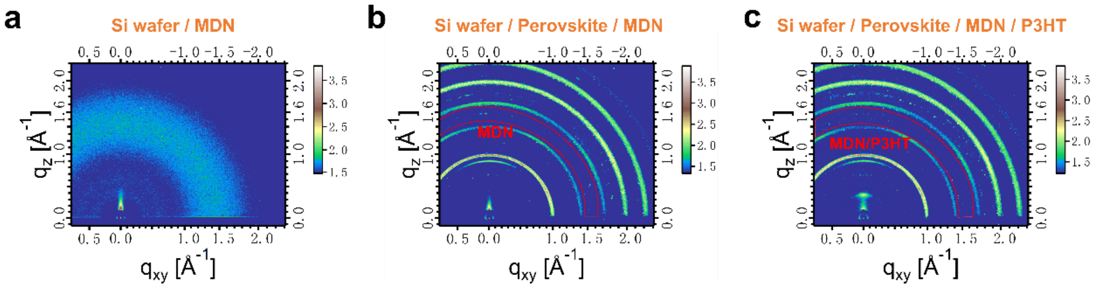

Supplementary Fig. 19. 2D GIWAXS patterns based on the structures of (**a**) Si-wafer/MDN, (**b**) Si-wafer/perovskite/MDN, and (**c**) Si-wafer/perovskite/MDN/P3HT.

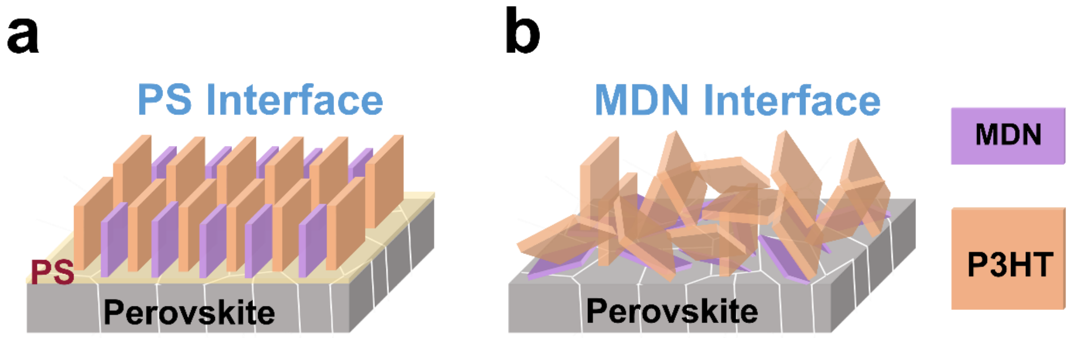

Supplementary Fig. 20. Schematic diagram of the molecular stacking with (**a**) PS interface and (**b**) MDN interface.

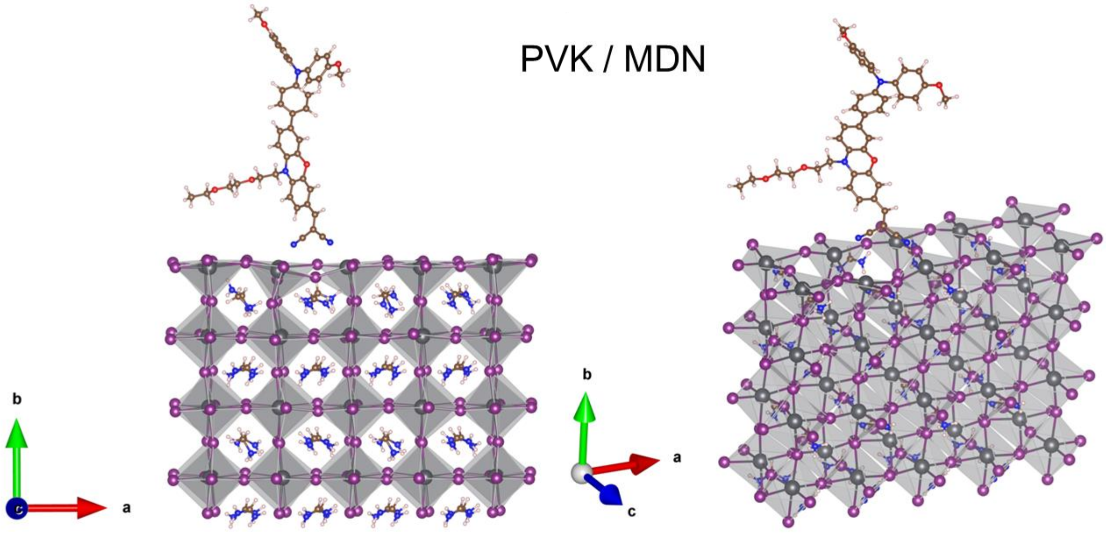

Supplementary Fig. 21. Snapshot of PVK/MDN.

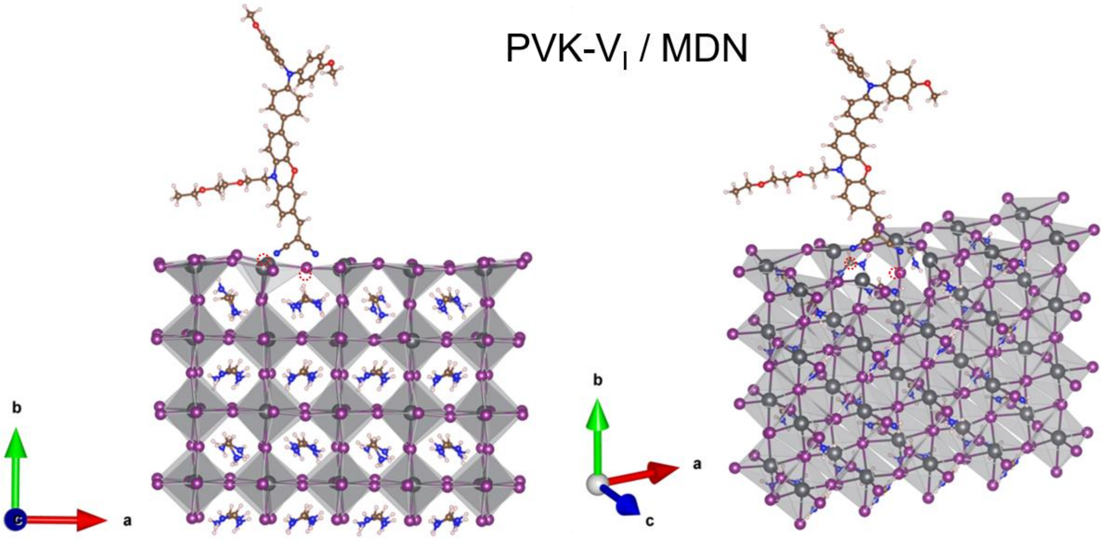

Supplementary Fig. 22. Snapshot of PVK-V_I/MDN.

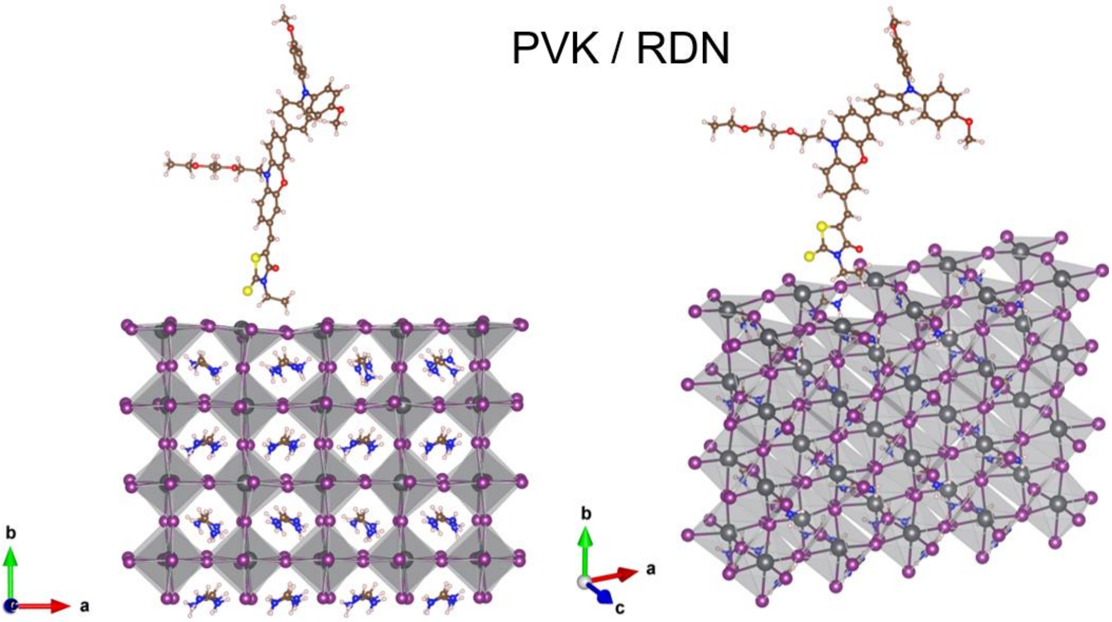

Supplementary Fig. 23. Snapshot of PVK/RDN.

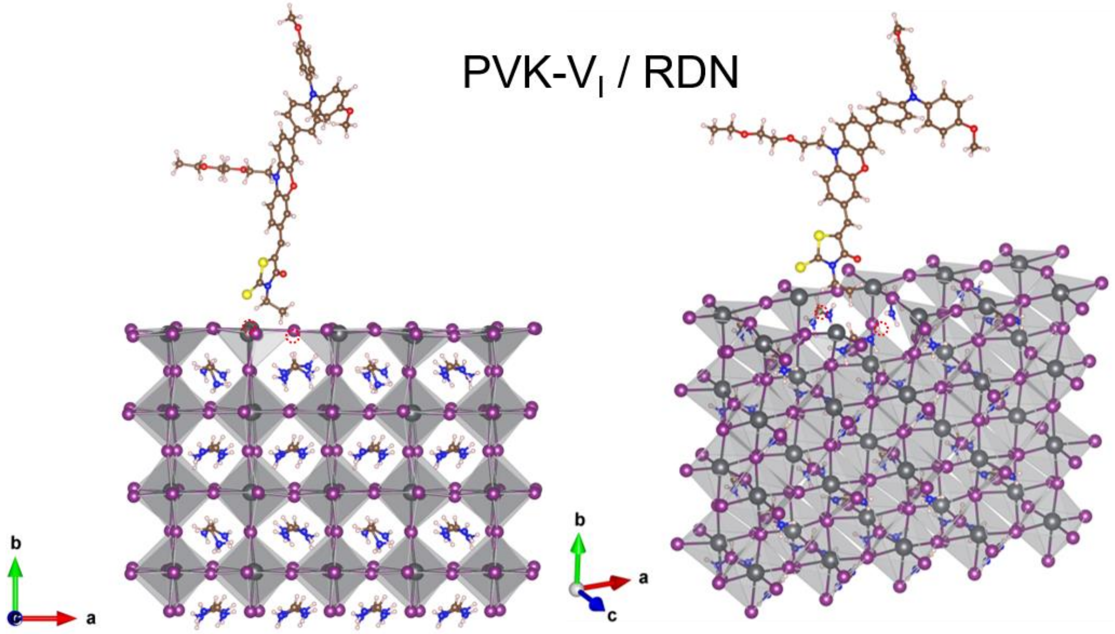

Supplementary Fig. 24. Snapshot of PVK-V_I/RDN.

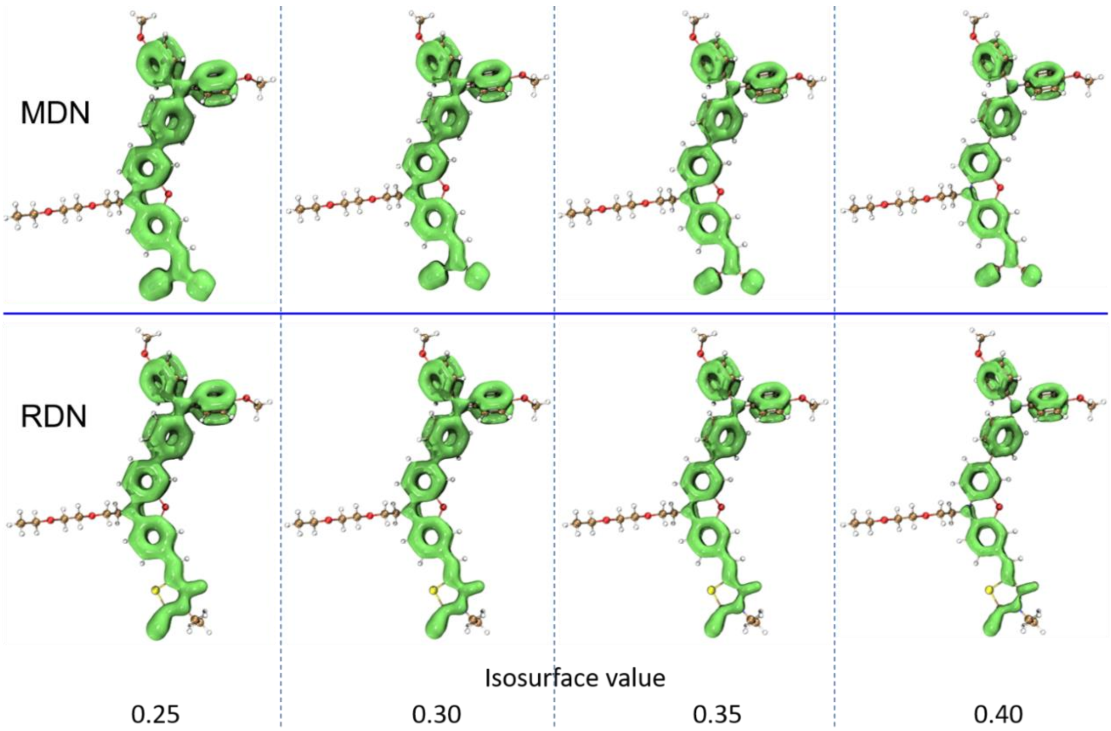

Supplementary Fig. 25. LOL-π maps of MDN and RDN at different isosurface values. The π orbitals are displayed in green.

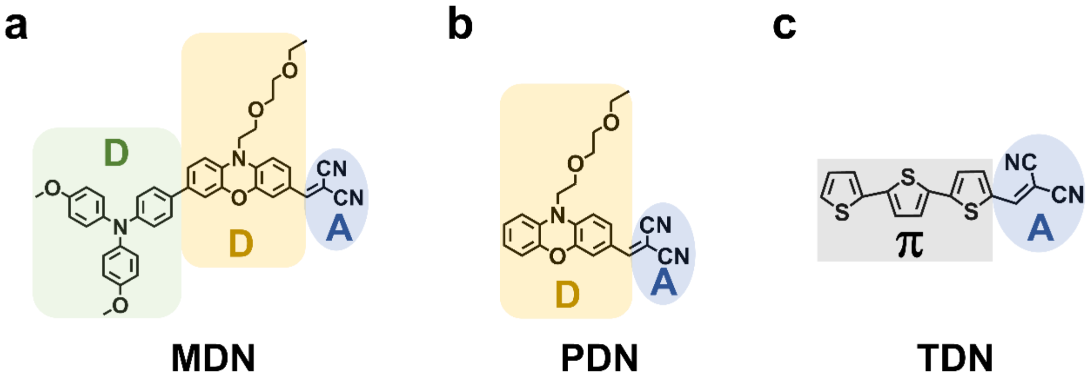

Supplementary Fig. 26. Molecular structure of (**a**) MDN, (**b**) PDN, (**c**) and TDN.

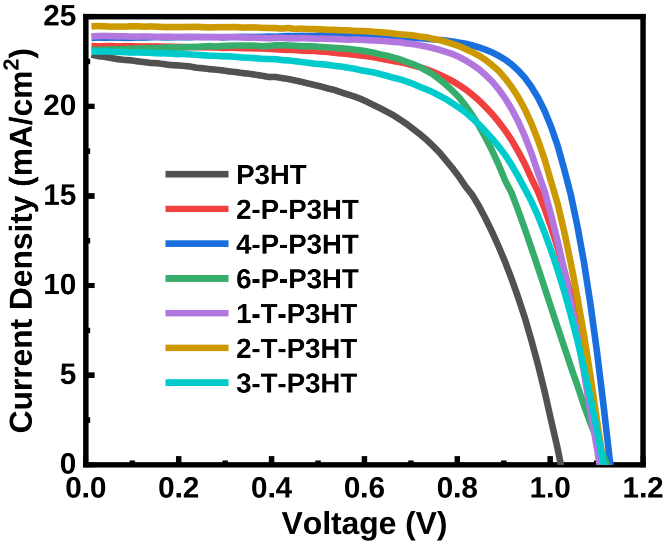

Supplementary Fig. 27. The *J*–*V* curves of the champion devices based on the different HTMs.

Supplementary Table 9. Photovoltaic parameters of champion devices based on different HTMs measured under AM 1.5G Illumination.

Device	V_{OC} (V)	J_{SC} ($mA \cdot cm^{-2}$)	*FF* (%)	*PCE* (%)
**P3HT**	1.02	23.07	56.41	13.29
**2-P-P3HT**	1.11	23.39	66.04	17.22
**4-P-P3HT**	1.13	23.84	76.10	20.47
**6-P-P3HT**	1.12	22.49	63.39	16.01
**1-T-P3HT**	1.11	23.92	70.82	18.73
**2-T-P3HT**	1.11	24.47	71.68	19.53
**3-T-P3HT**	1.11	23.08	62.62	16.09

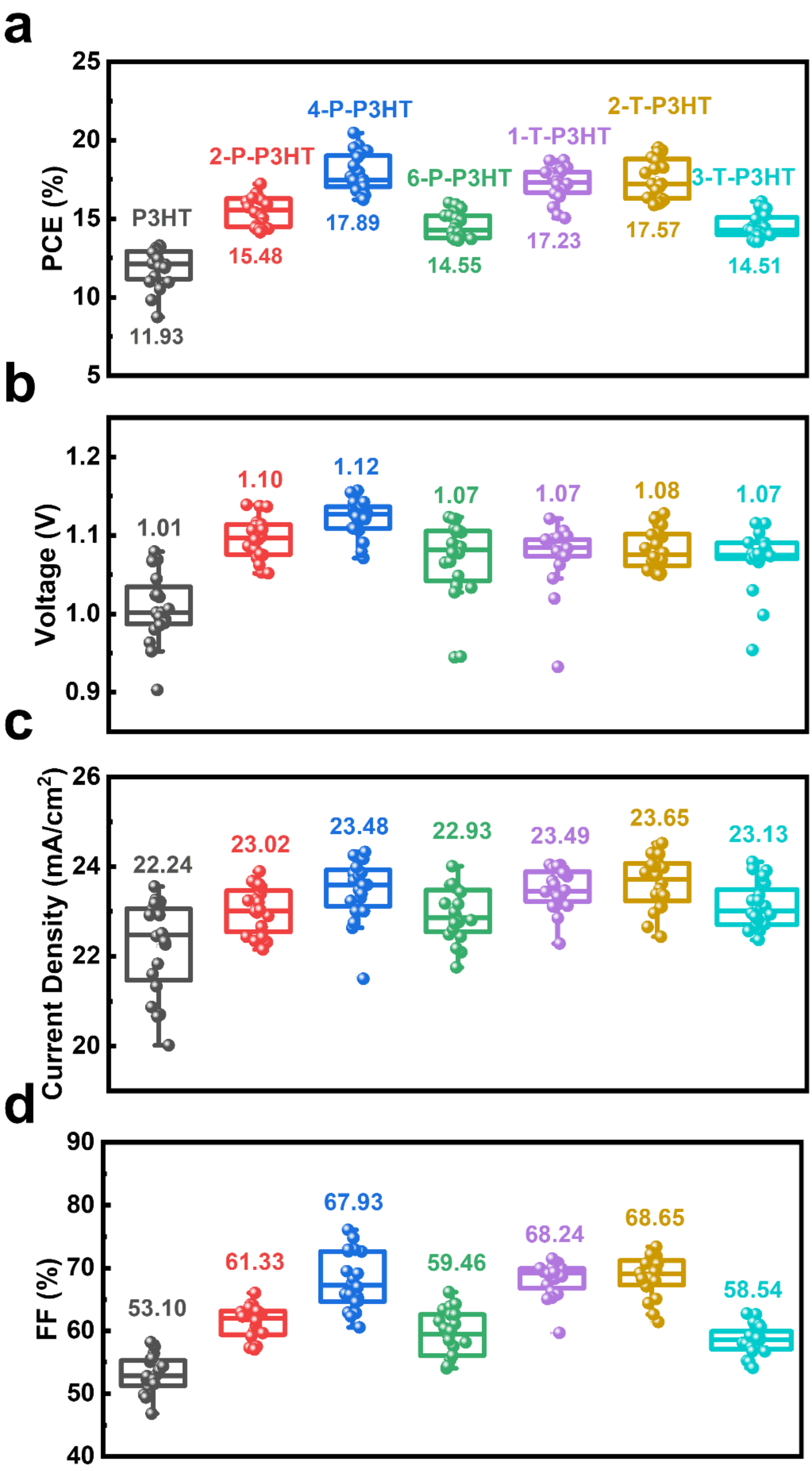

Supplementary Fig. 28. The statistical distributions of 20 devices with different HTMs of (**a**) *PCE*, (**b**) V_{OC}, (**c**) J_{SC}, (**d**) *FF*.

Supplementary Table 10. Comparison of the device performance obtained with different HTMs. The average values and standard deviations are calculated from 20 devices fabricated at the same conditions.

Device	V_{OC} (V)	J_{SC} (mA·cm^{-2})	*FF* (%)	*PCE* (%)
**P3HT**	1.01 ± 0.04	22.24 ± 1.04	53.10 ± 2.90	11.93 ± 1.22
**2-P-P3HT**	1.10 ± 0.03	23.02 ± 0.52	61.33 ± 2.66	15.48 ± 0.96
**4-P-P3HT**	1.12 ± 0.02	23.48 ± 0.68	67.93 ± 4.51	17.89 ± 1.23
**6-P-P3HT**	1.07 ± 0.05	22.93 ± 0.58	59.46 ± 3.70	14.55 ± 0.81
**1-T-P3HT**	1.07 ± 0.04	23.49 ± 0.45	68.24 ± 2.74	17.23 ± 1.05
**2-T-P3HT**	1.08 ± 0.03	23.65 ± 0.59	68.65 ± 3.27	17.57 ± 1.28
**3-T-P3HT**	1.07 ± 0.04	23.13 ± 0.51	58.54 ± 2.36	14.51 ± 0.79

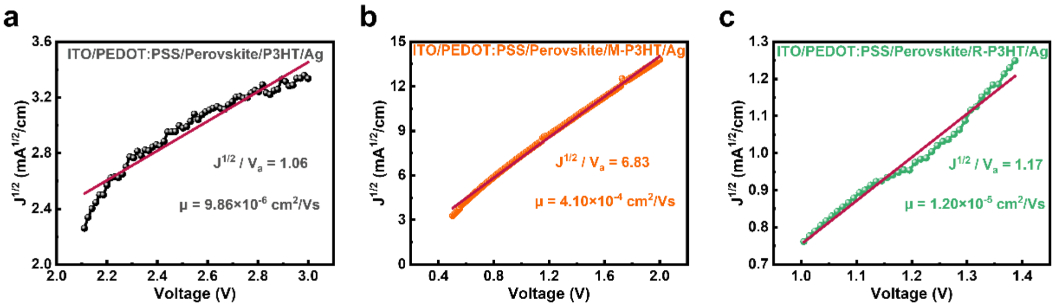

Supplementary Fig. 29. Hole mobility measurement characteristics by the space charge limited current (SCLC) method for the devices of (**a**) ITO/PEDOT:PSS/perovskite/P3HT/Ag, (**b**) ITO/PEDOT:PSS/perovskite/M-/P3HT/Ag, (**c**) ITO/PEDOT:PSS/perovskite/R-P3HT/Ag.

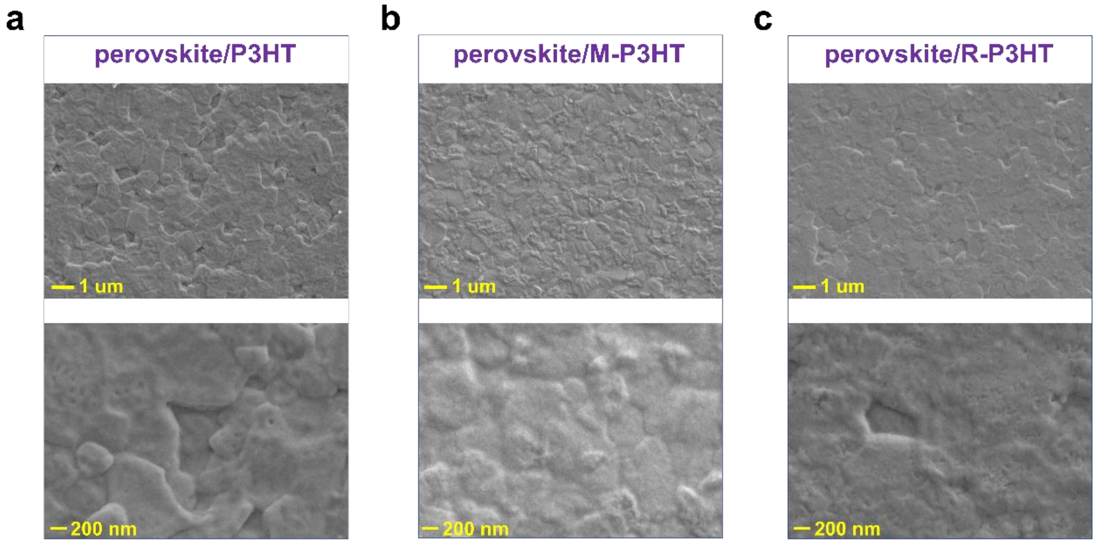

Supplementary Fig. 30. The top view SEM images with aging devices of (**a**) perovskite/P3HT, (**b**) perovskite/M-P3HT, and (**c**) perovskite/R-P3HT.

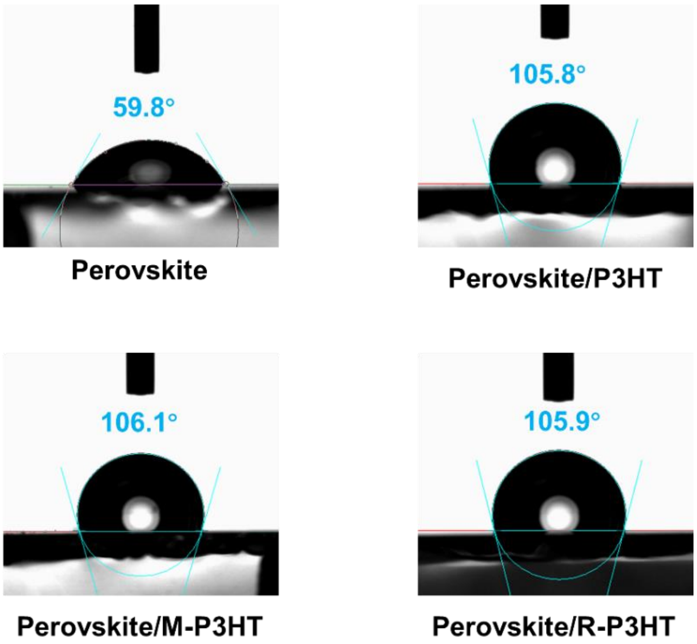

Supplementary Fig. 31. The water contact angle of perovskite with or without different HTMs.

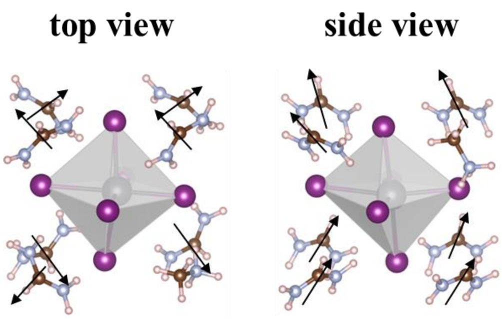

Supplementary Fig. 32. Primitive cell of $FA_{0.875}MA_{0.125}PbI_3$. Only one Pb–I octahedron is displayed for clarity. The arrows denote the orientations of C–H bond in FA molecule.

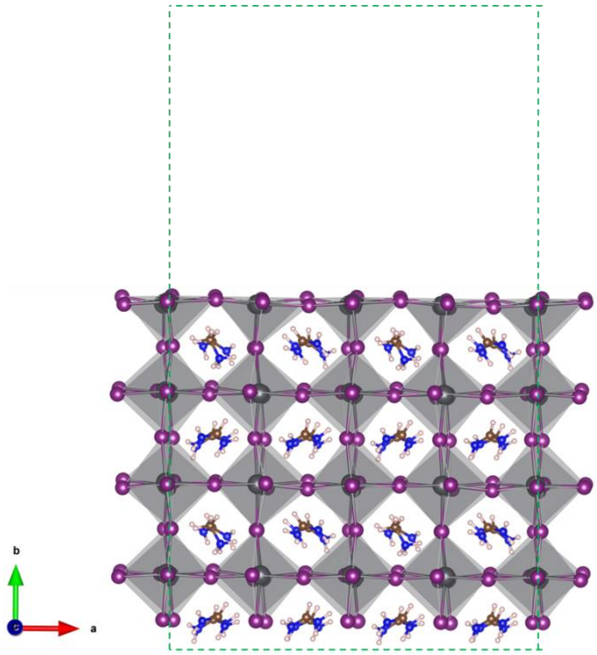

Supplementary Fig. 33. Slab model with a vacuum of 20 Å of $FA_{0.875}MA_{0.125}PbI_3$ after structural optimization.

^1H NMR, ^{13}C NMR and MS spectra

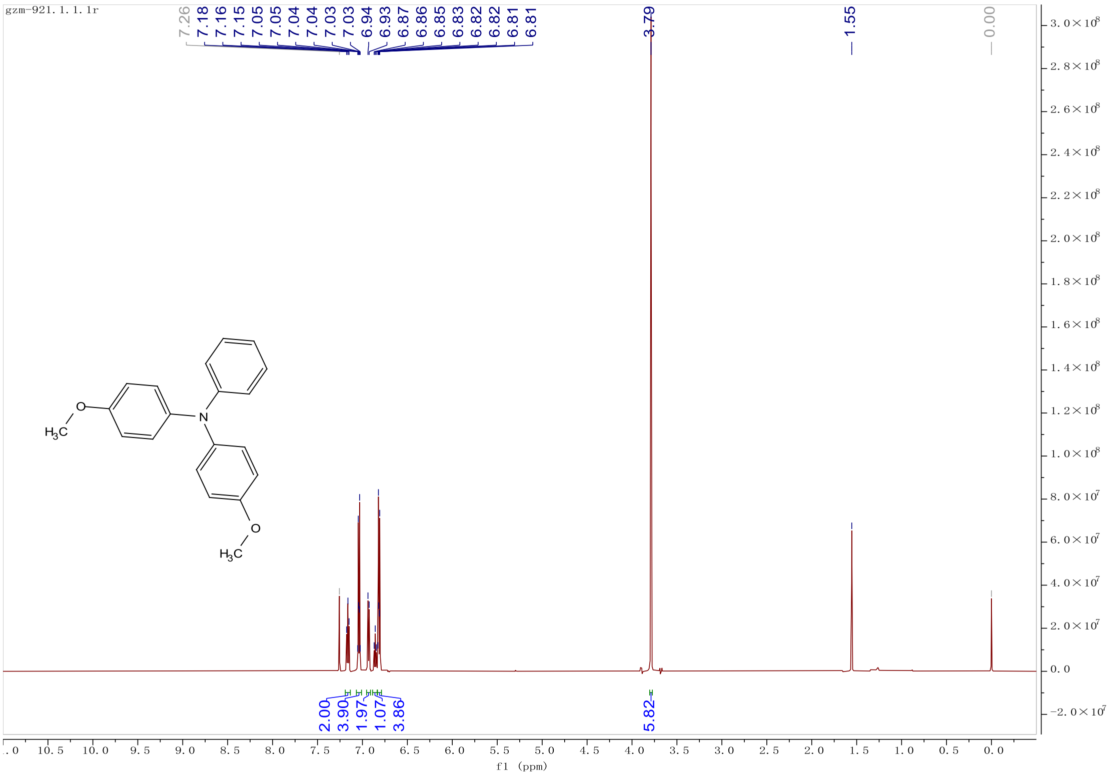

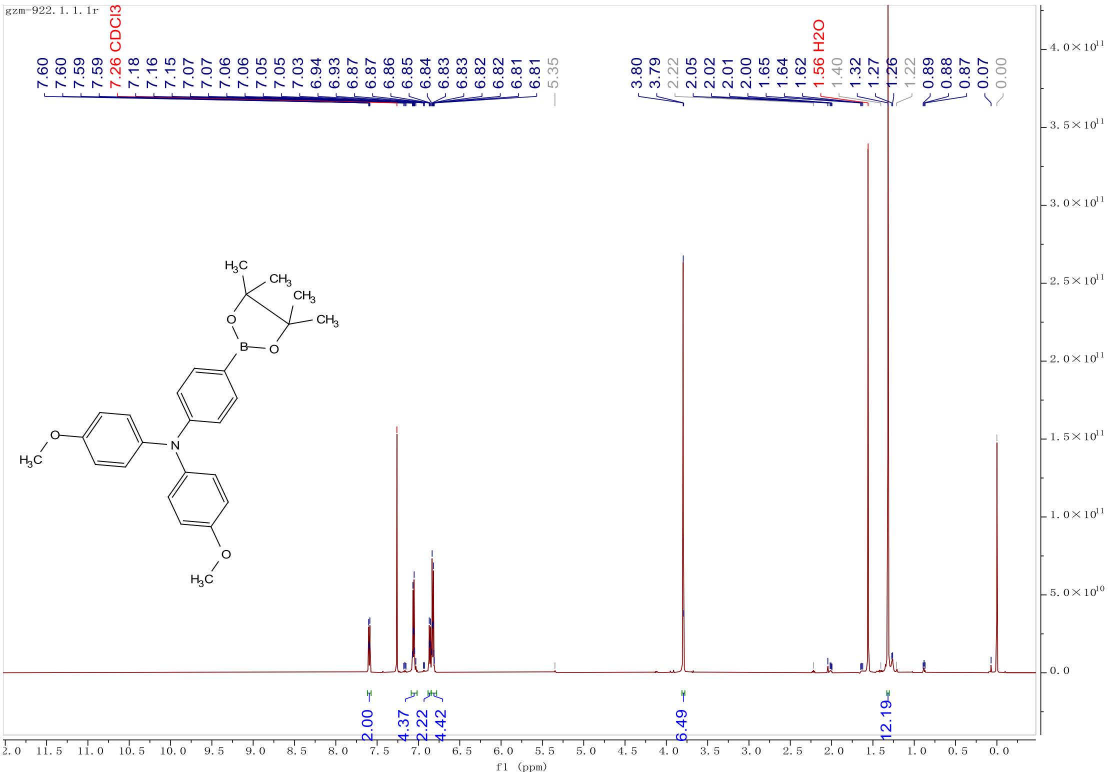

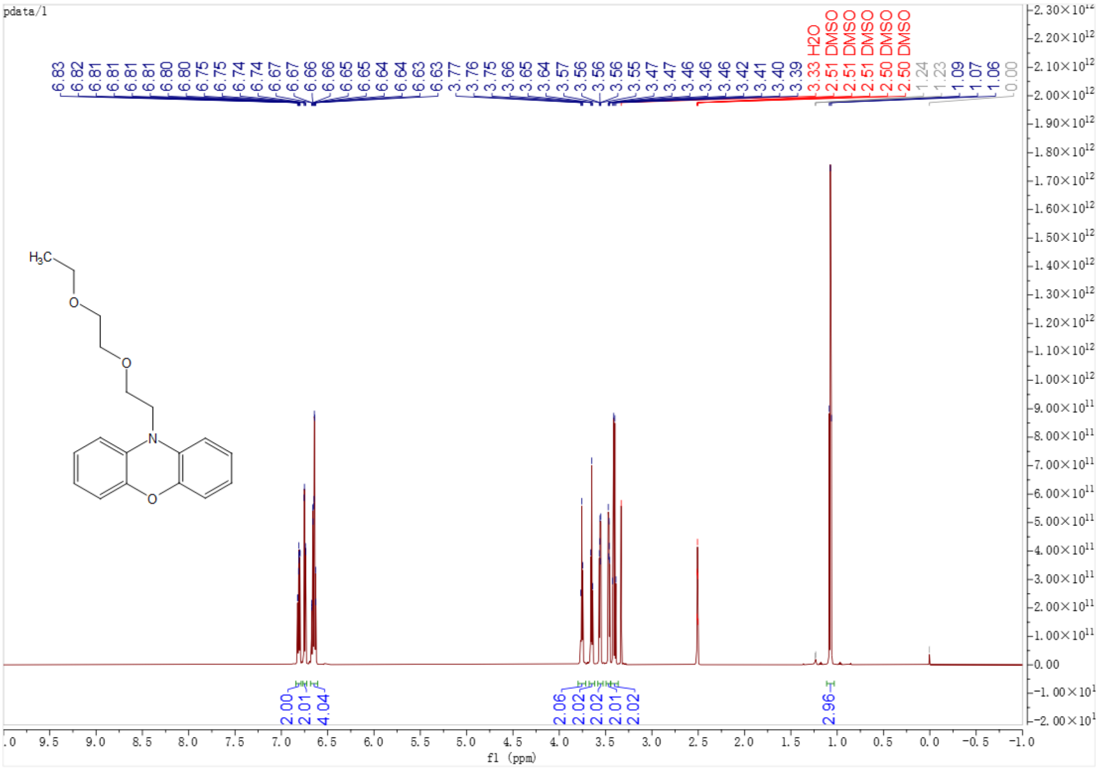
pdata/1
6.83
6.82
6.81
6.81
6.81
6.81
6.80
6.80
6.75
6.75
6.74
6.74
6.67
6.67
6.66
6.66
6.65
6.65
6.64
6.64
6.63
6.63
3.77
3.76
3.75
3.66
3.65
3.64
3.57
3.56
3.56
3.56
3.55
3.47
3.47
3.46
3.46
3.46
3.42
3.41
3.40
3.39
3.33 H2O
2.51 DMSO
2.51 DMSO
2.51 DMSO
2.50 DMSO
2.50 DMSO
1.24
1.23
1.09
1.07
1.06
0.00
2.00
2.01
4.04
2.06
2.02
2.02
2.01
2.02
2.96
f1 (ppm)

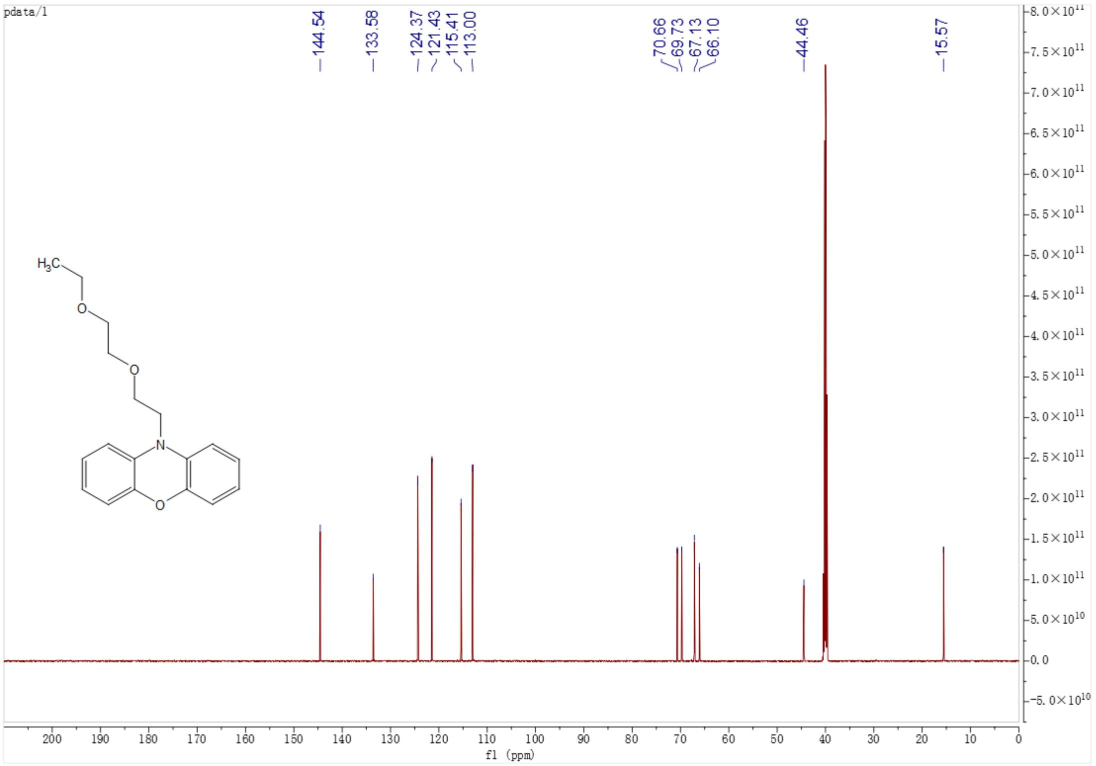
pdata/1
144.54
133.58
124.37
121.43
115.41
113.00
70.66
69.73
67.13
66.10
44.46
15.57
f1 (ppm)

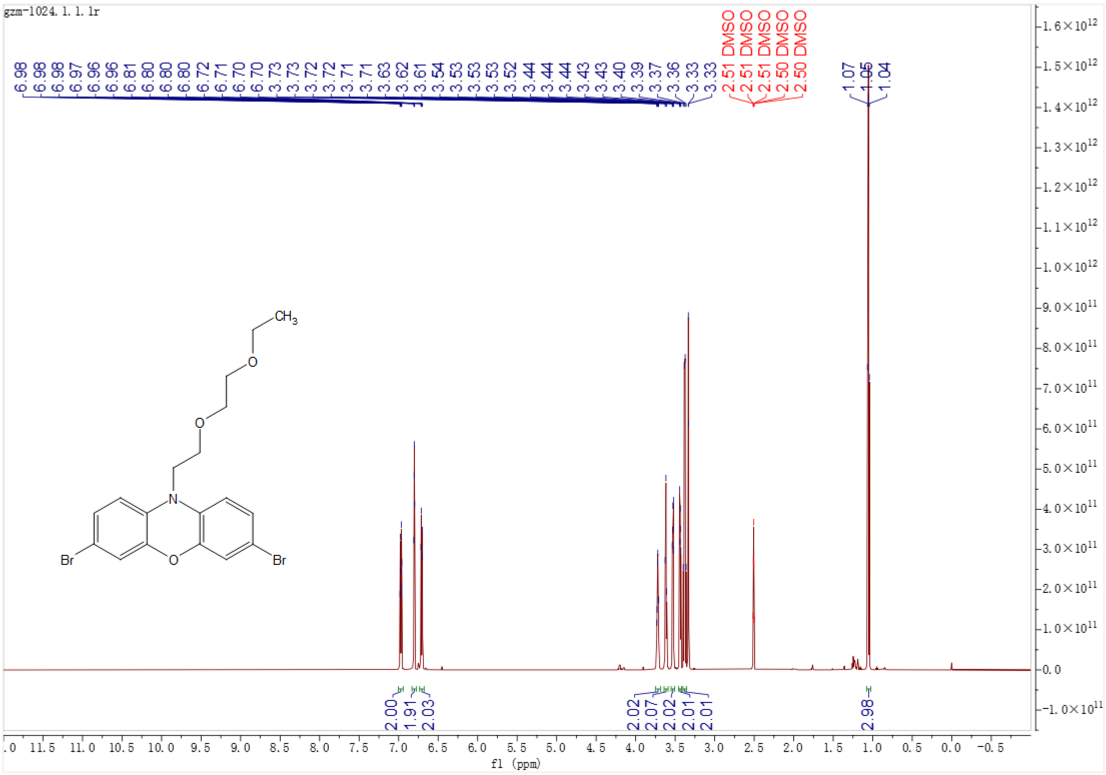
gzm-1024.1.1.1r
6.98
6.98
6.98
6.97
6.96
6.96
6.81
6.80
6.80
6.80
6.72
6.71
6.70
6.70
3.73
3.73
3.72
3.72
3.71
3.63
3.62
3.61
3.54
3.53
3.53
3.53
3.52
3.44
3.44
3.44
3.43
3.43
3.40
3.39
3.37
3.36
3.33
2.51 DMSO
2.51 DMSO
2.51 DMSO
2.50 DMSO
2.50 DMSO
1.07
1.05
1.04
CH3
O
O
N
O
Br
Br
2.00
1.91
2.03
2.02
2.07
2.02
2.01
2.01
2.98
f1 (ppm)

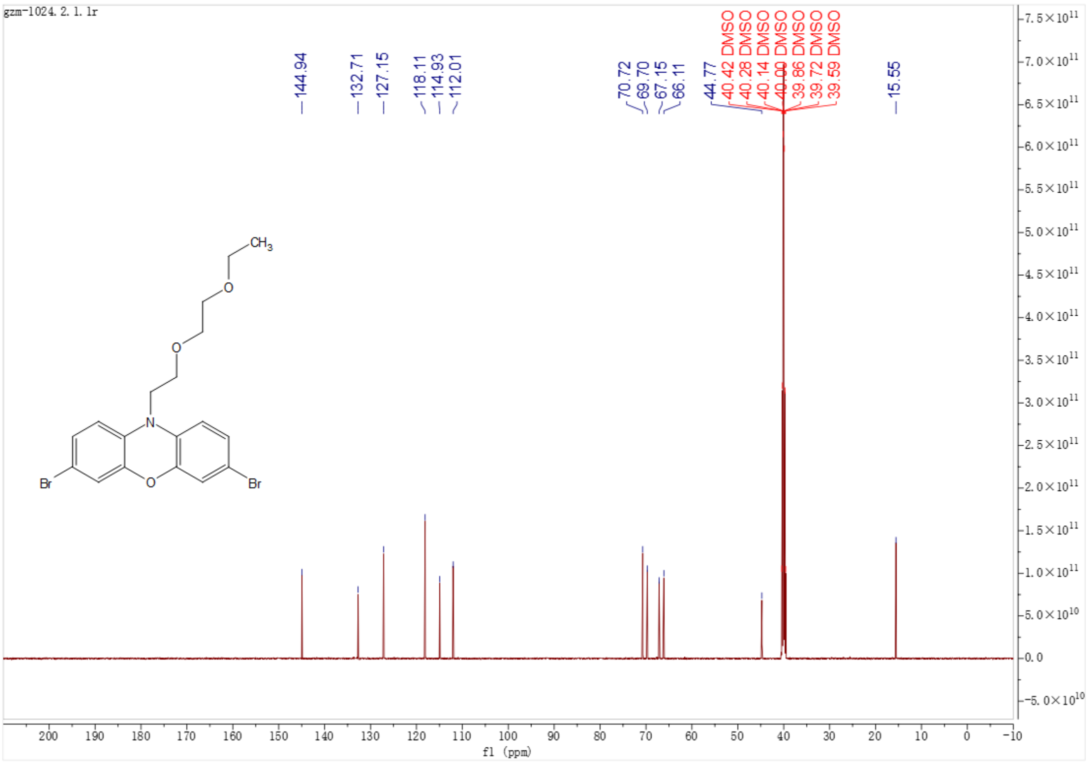
gzm-1024.2.1.1r
144.94
132.71
127.15
118.11
114.93
112.01
70.72
69.70
67.15
66.11
44.77
40.42 DMSO
40.28 DMSO
40.14 DMSO
40.00 DMSO
39.86 DMSO
39.72 DMSO
39.59 DMSO
15.55
CH3
O
O
N
O
Br
Br
f1 (ppm)

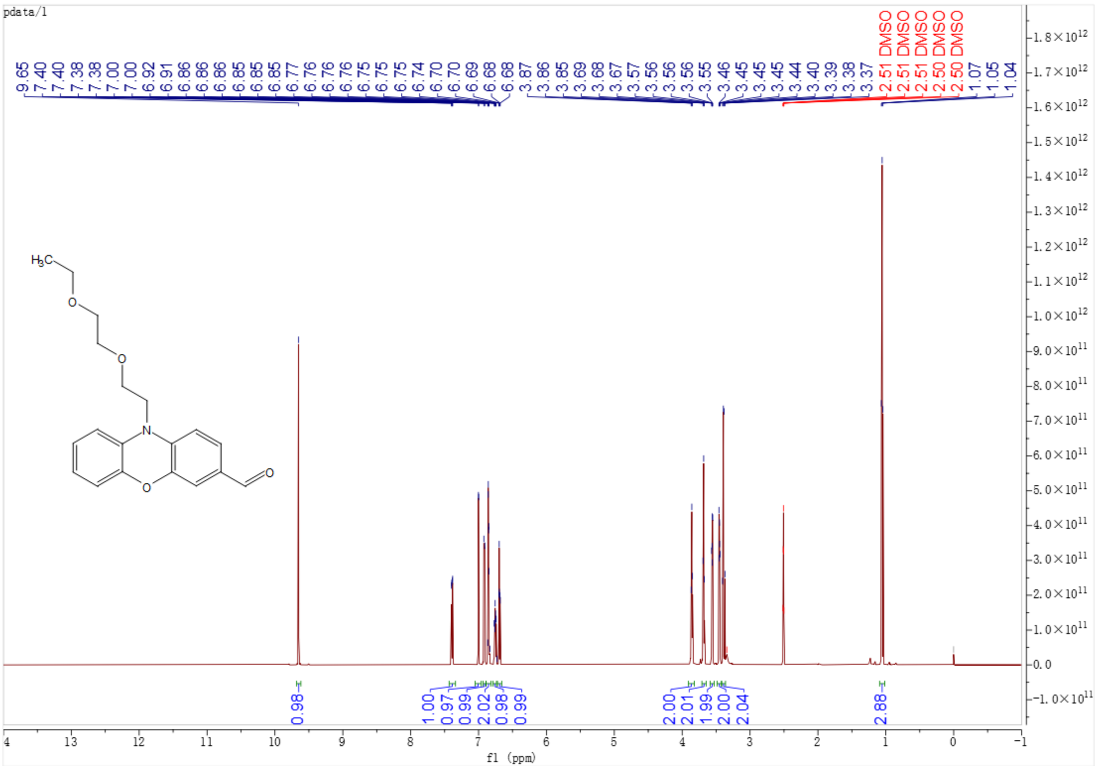

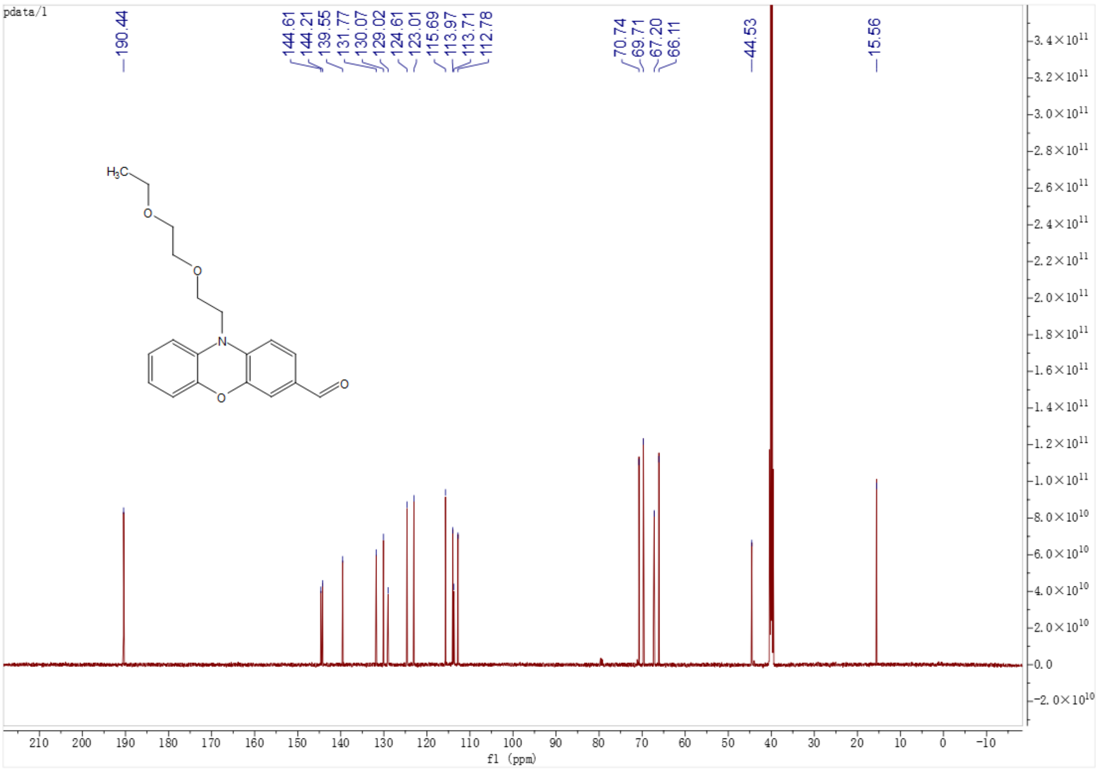

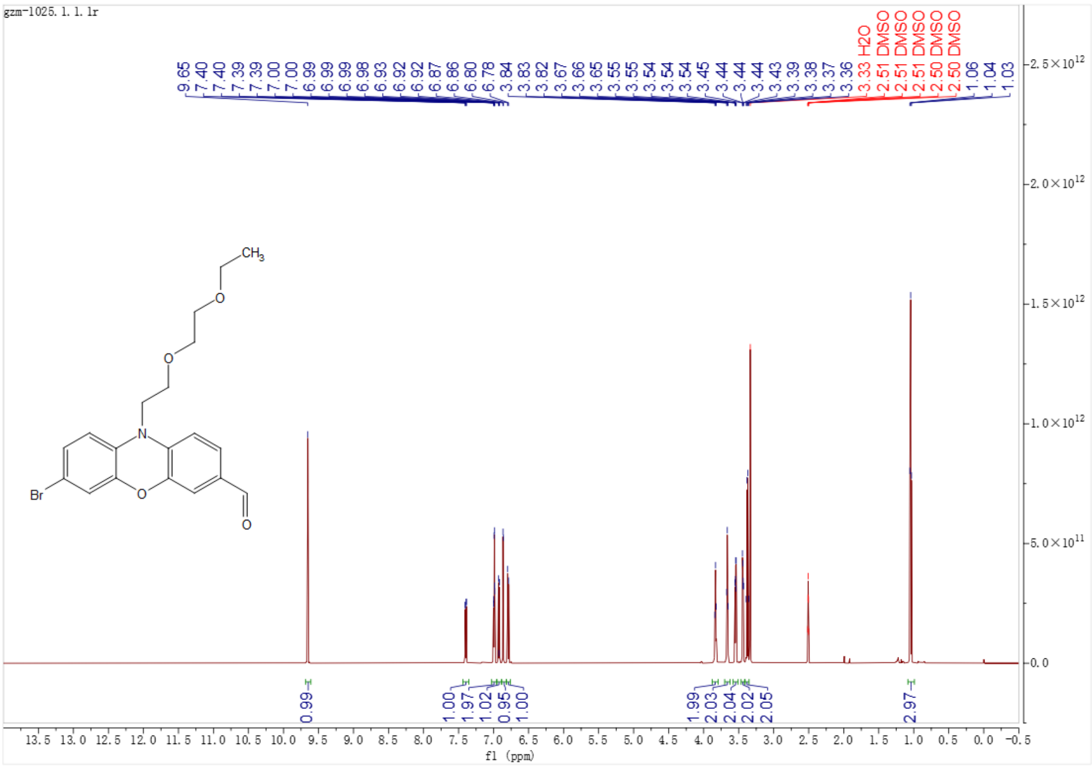

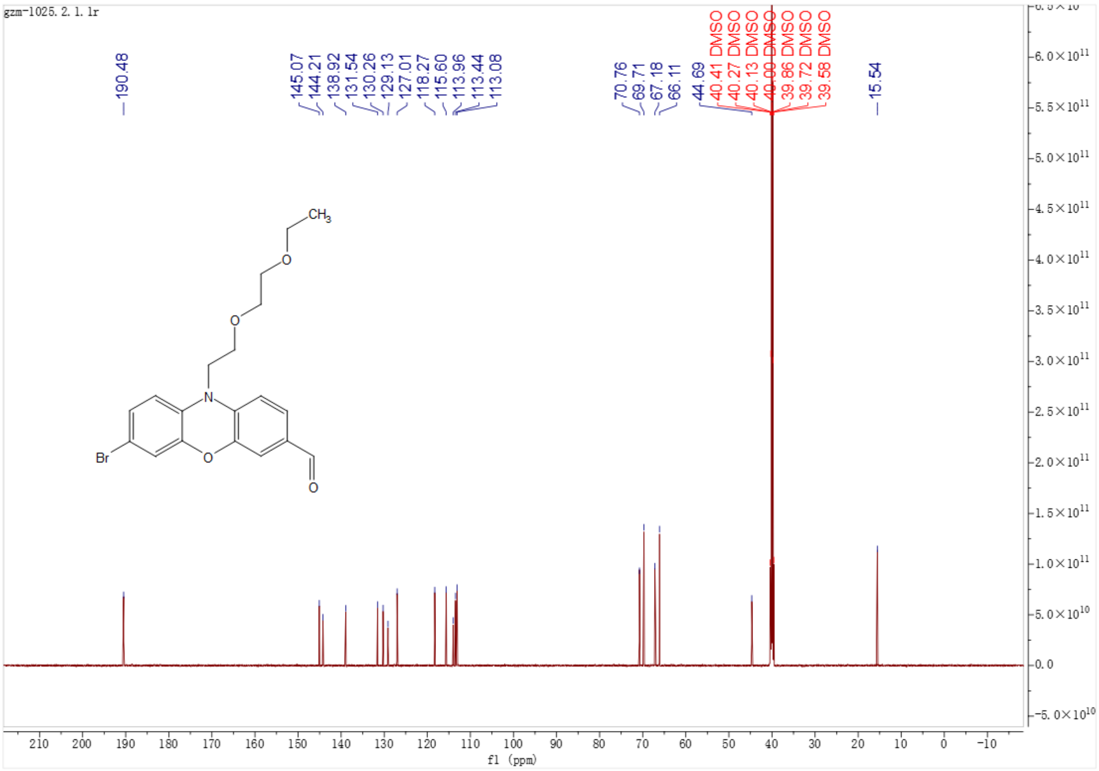

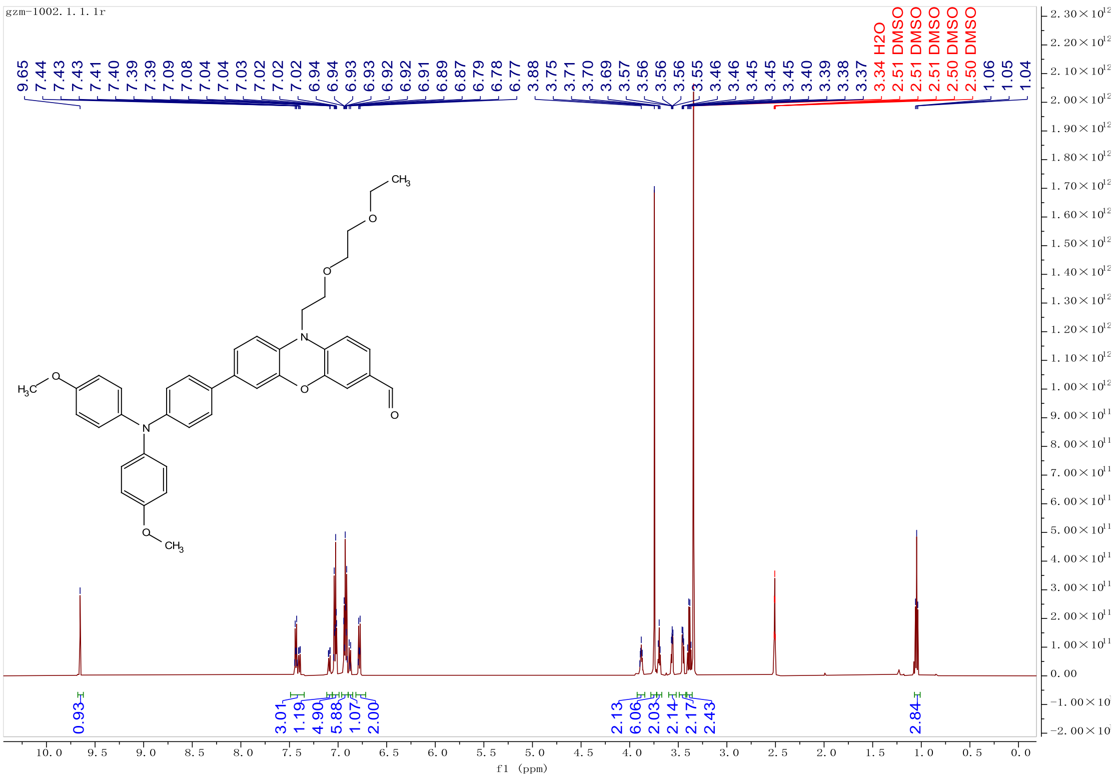
gzm-1002.1.1.1r
9.65 7.44 7.43 7.41 7.40 7.39 7.39 7.09 7.08 7.04 7.04 7.03 7.02 7.02 7.02 6.94 6.94 6.93 6.93 6.92 6.92 6.91 6.89 6.87 6.79 6.78 6.77 3.88 3.75 3.71 3.70 3.69 3.57 3.56 3.56 3.56 3.55 3.46 3.45 3.45 3.40 3.39 3.38 3.37
3.34 H2O
2.51 DMSO
2.51 DMSO
2.51 DMSO
2.50 DMSO
2.50 DMSO
1.06 1.05 1.04
f1 (ppm)

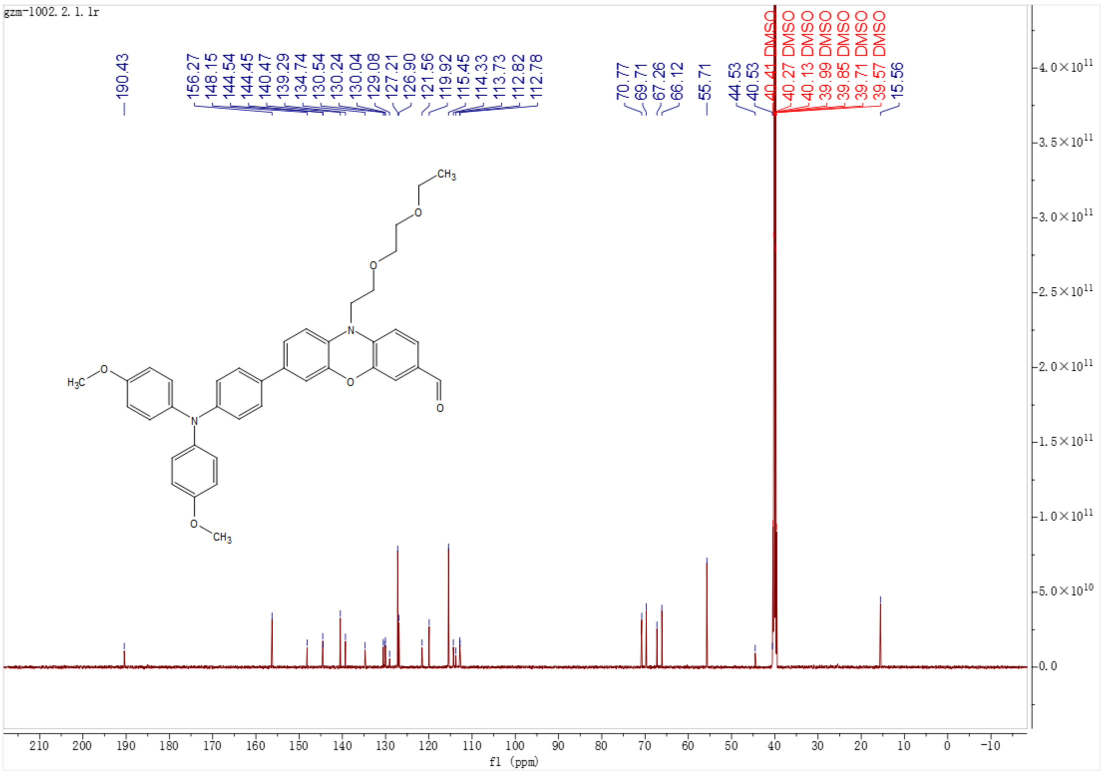
gzm-1002.2.1.1r
190.43 156.27 148.15 144.54 144.45 140.47 139.29 134.74 130.54 130.24 130.04 129.08 127.21 126.90 121.56 119.92 115.45 114.33 113.73 112.82 112.78 70.77 69.71 67.26 66.12 55.71 44.53 40.53
40.41 DMSO
40.27 DMSO
40.13 DMSO
39.99 DMSO
39.85 DMSO
39.71 DMSO
39.57 DMSO
15.56
f1 (ppm)

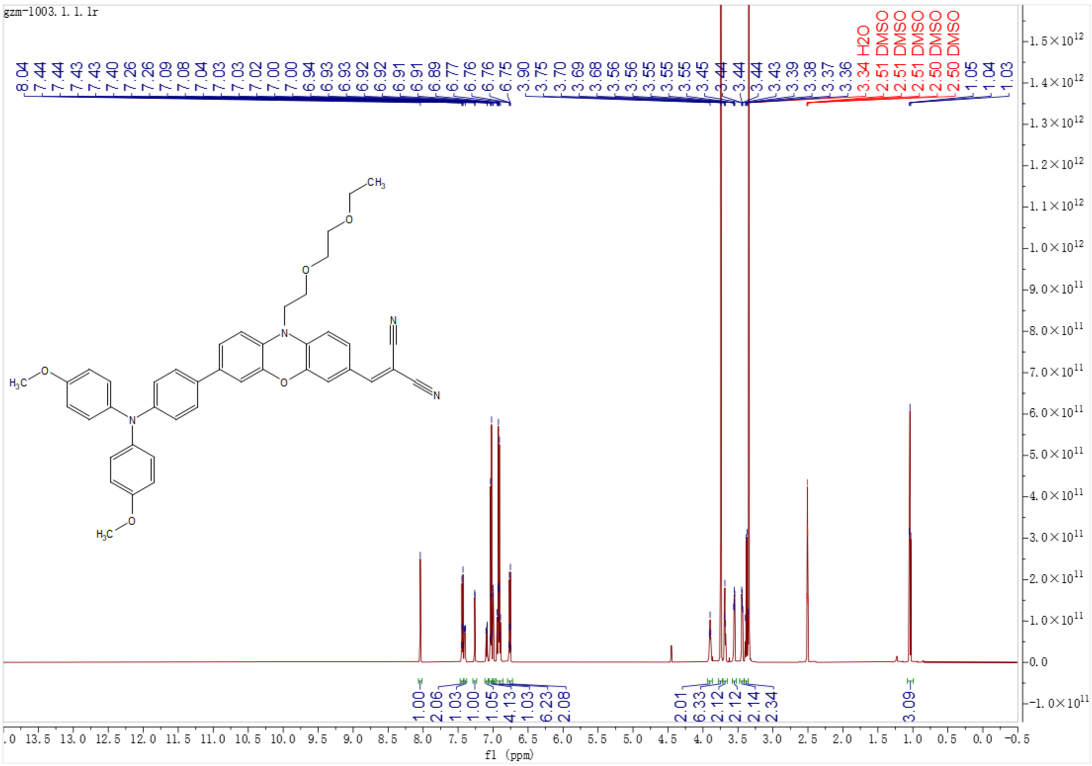

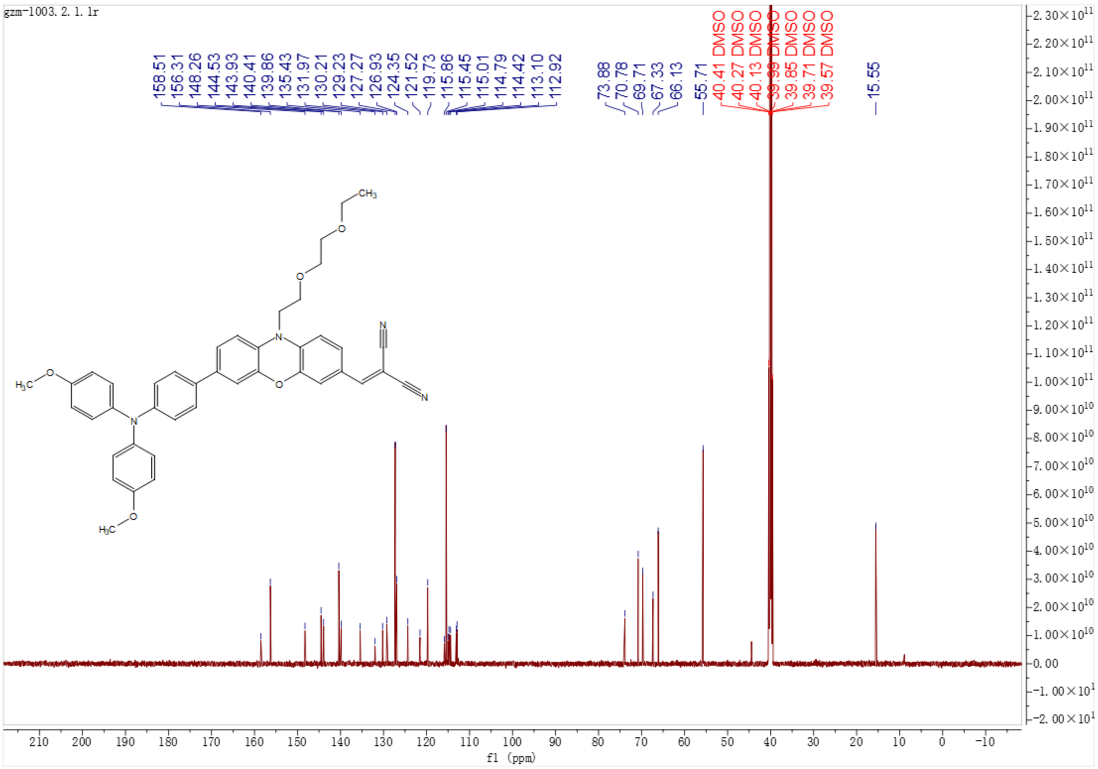

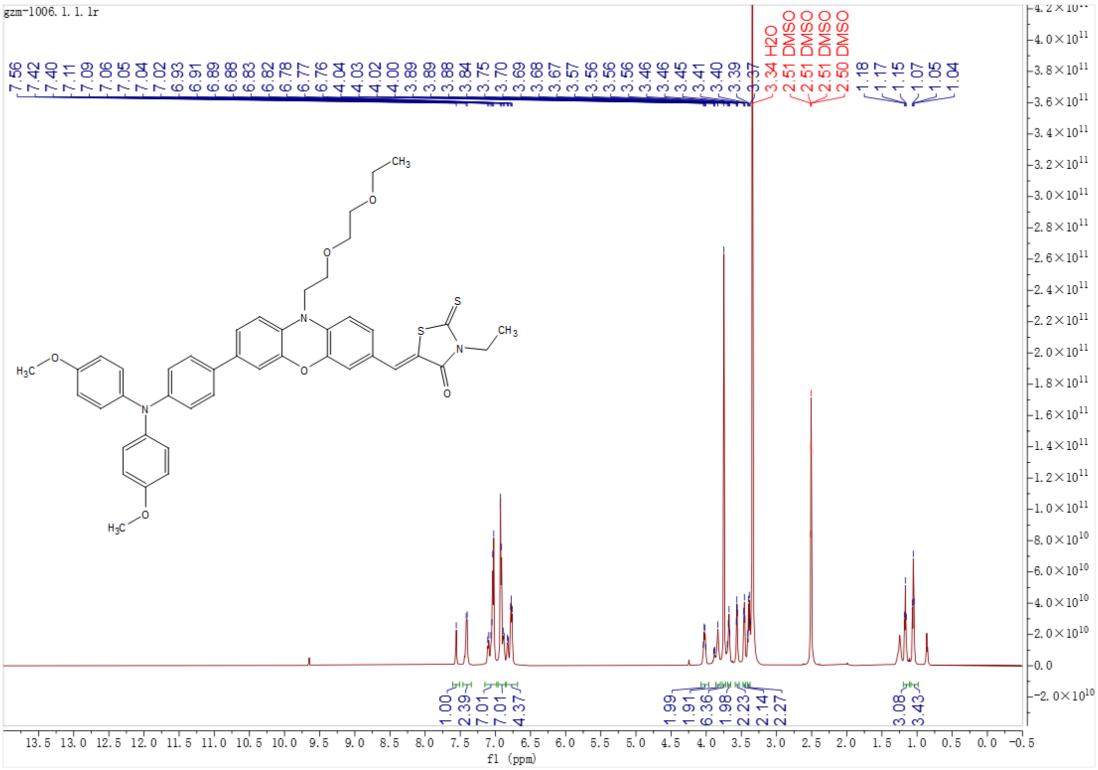

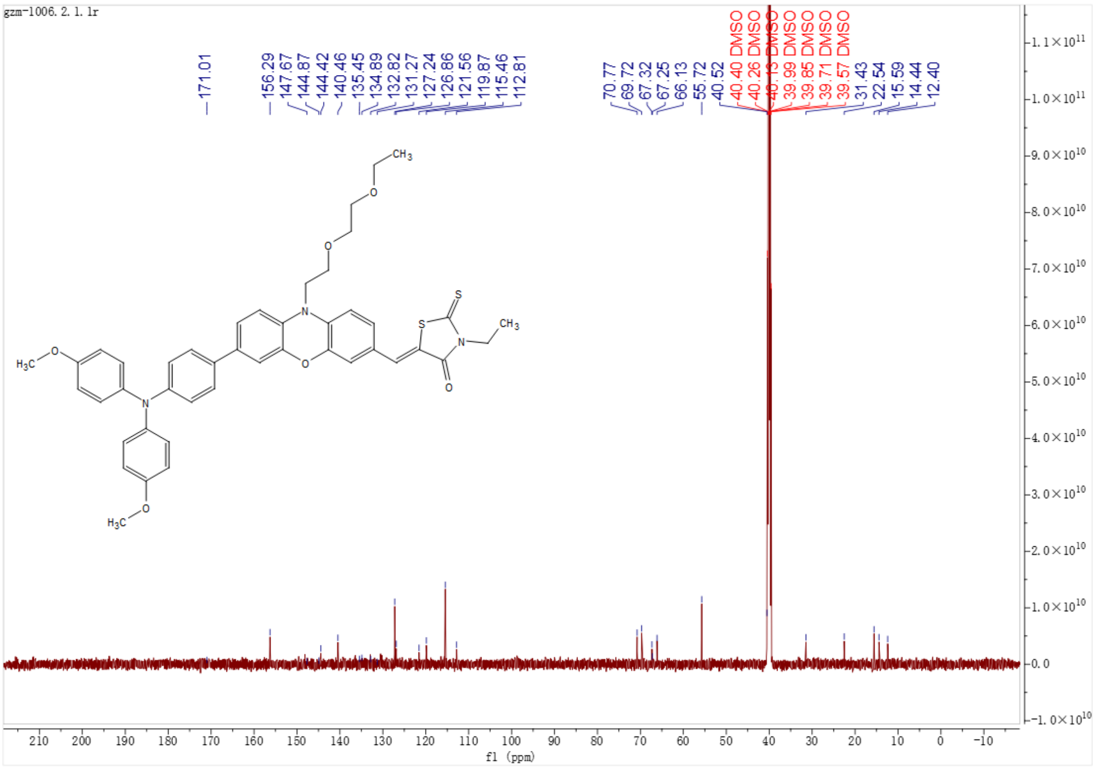

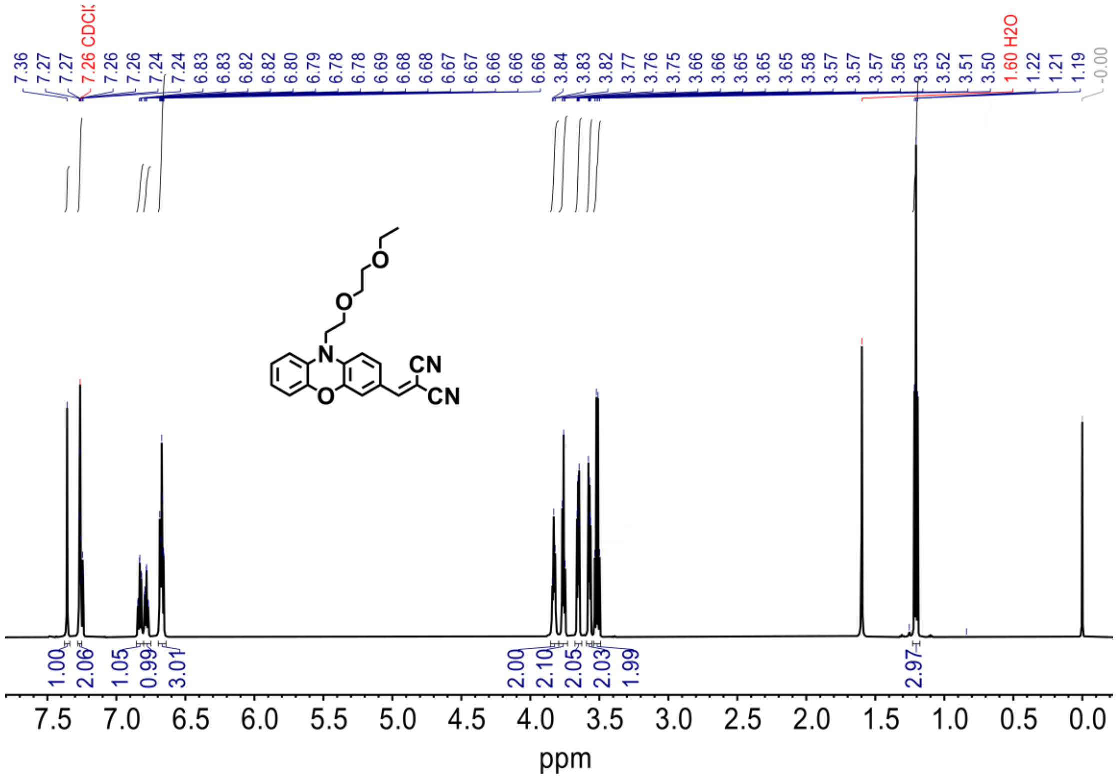
7.36
7.27
7.27
7.26 CDCl3
7.26
7.26
7.24
7.24
6.83
6.83
6.82
6.82
6.80
6.79
6.78
6.78
6.69
6.68
6.68
6.67
6.67
6.66
6.66
6.66
3.84
3.83
3.82
3.77
3.76
3.75
3.66
3.66
3.65
3.65
3.65
3.58
3.57
3.57
3.57
3.56
3.53
3.52
3.51
3.50
1.60 H2O
1.22
1.21
1.19
-0.00
N
O
O
O
CN
CN
1.00
2.06
1.05
0.99
3.01
2.00
2.10
2.05
2.03
1.99
2.97
7.5 7.0 6.5 6.0 5.5 5.0 4.5 4.0 3.5 3.0 2.5 2.0 1.5 1.0 0.5 0.0
ppm

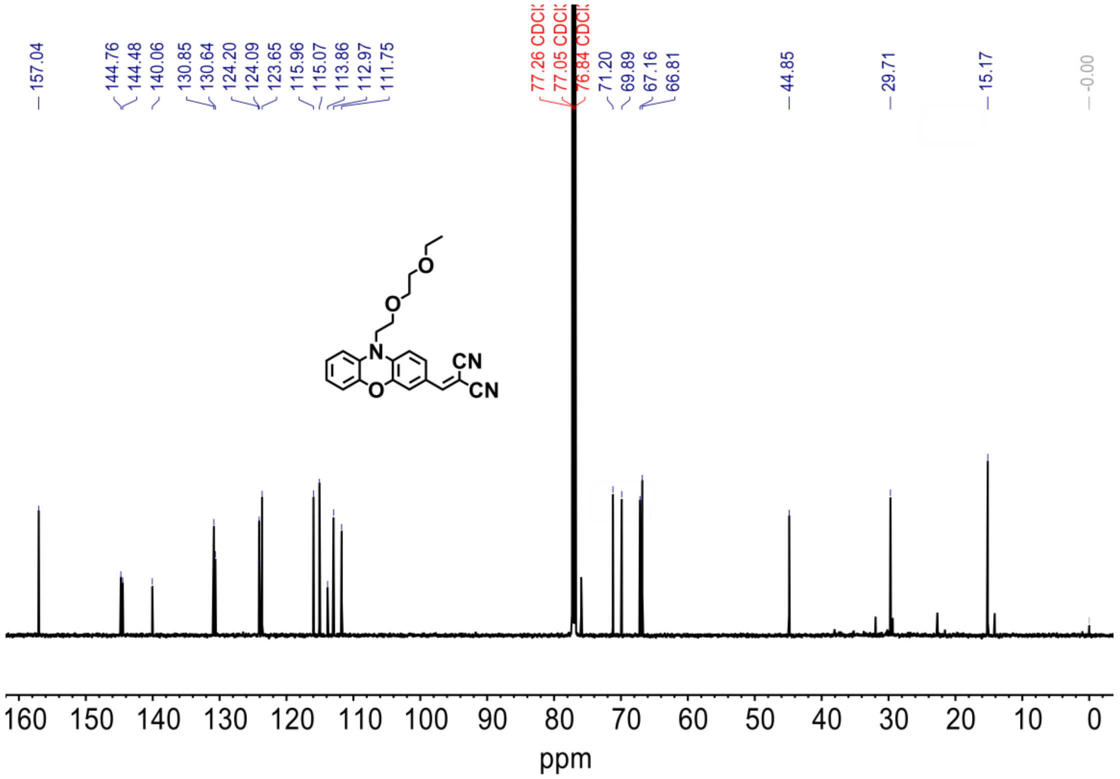
157.04
144.76
144.48
140.06
130.85
130.64
124.20
124.09
123.65
115.96
115.07
113.86
112.97
111.75
77.26 CDCl3
77.05 CDCl3
76.84 CDCl3
71.20
69.89
67.16
66.81
44.85
29.71
15.17
-0.00
N
O
O
O
CN
CN
160 150 140 130 120 110 100 90 80 70 60 50 40 30 20 10 0
ppm

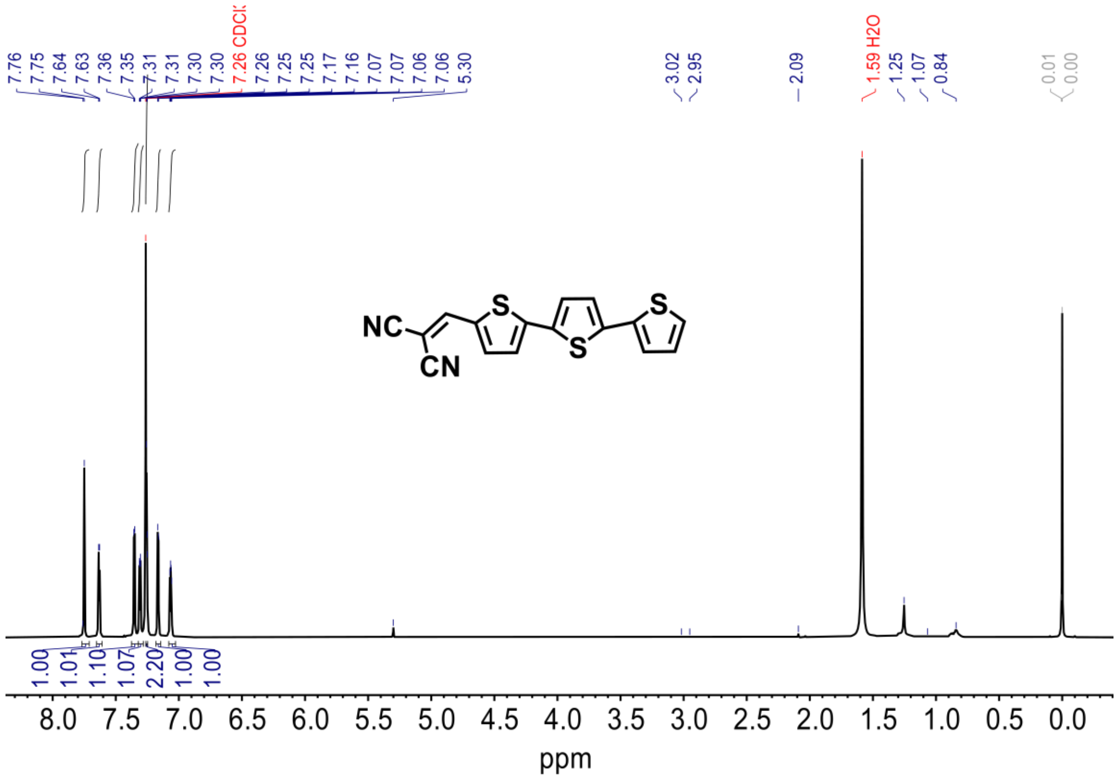
7.76 7.75 7.64 7.63 7.36 7.35 7.31 7.31 7.30 7.30 7.26 CDCl3 7.26 7.25 7.25 7.17 7.16 7.07 7.07 7.06 7.06 5.30
3.02 2.95
2.09
1.59 H2O 1.25 1.07 0.84
0.01 0.00
NC
CN
S
1.00 1.01 1.10 1.07 2.20 1.00 1.00
8.0 7.5 7.0 6.5 6.0 5.5 5.0 4.5 4.0 3.5 3.0 2.5 2.0 1.5 1.0 0.5 0.0
ppm

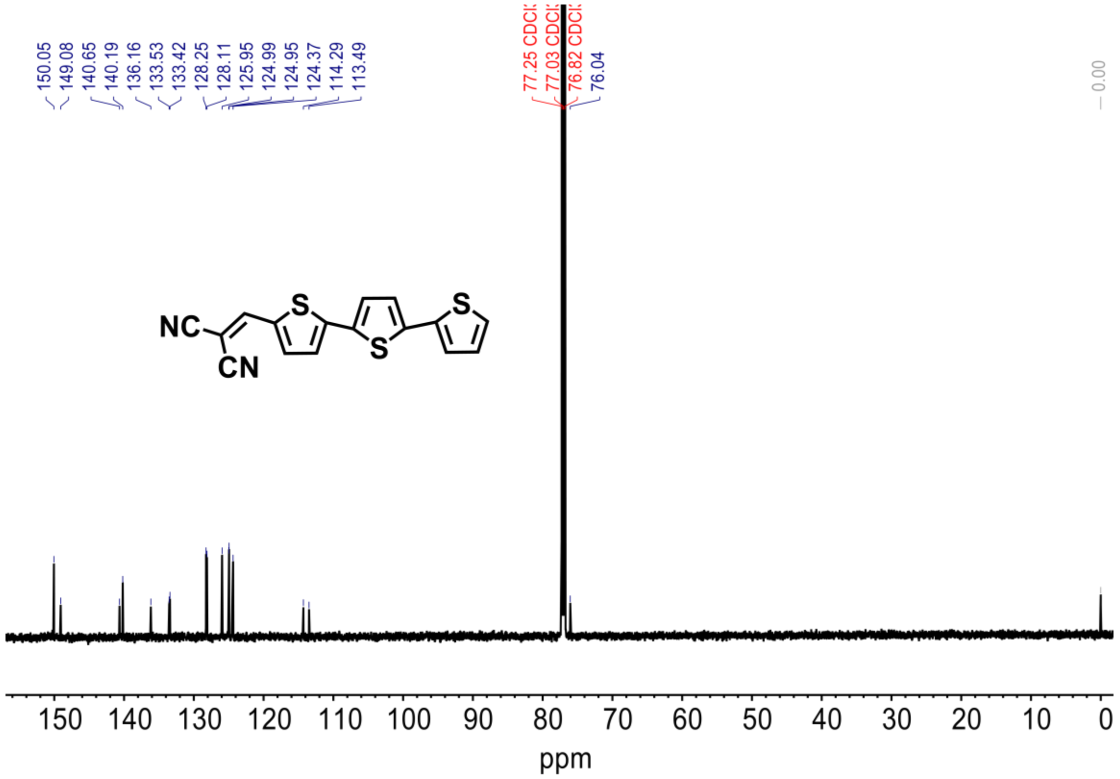
150.05 149.08 140.65 140.19 136.16 133.53 133.42 128.25 128.11 125.95 124.99 124.95 124.37 114.29 113.49
77.25 CDCl3 77.03 CDCl3 76.82 CDCl3 76.04
0.00
NC
CN
S
150 140 130 120 110 100 90 80 70 60 50 40 30 20 10 0
ppm

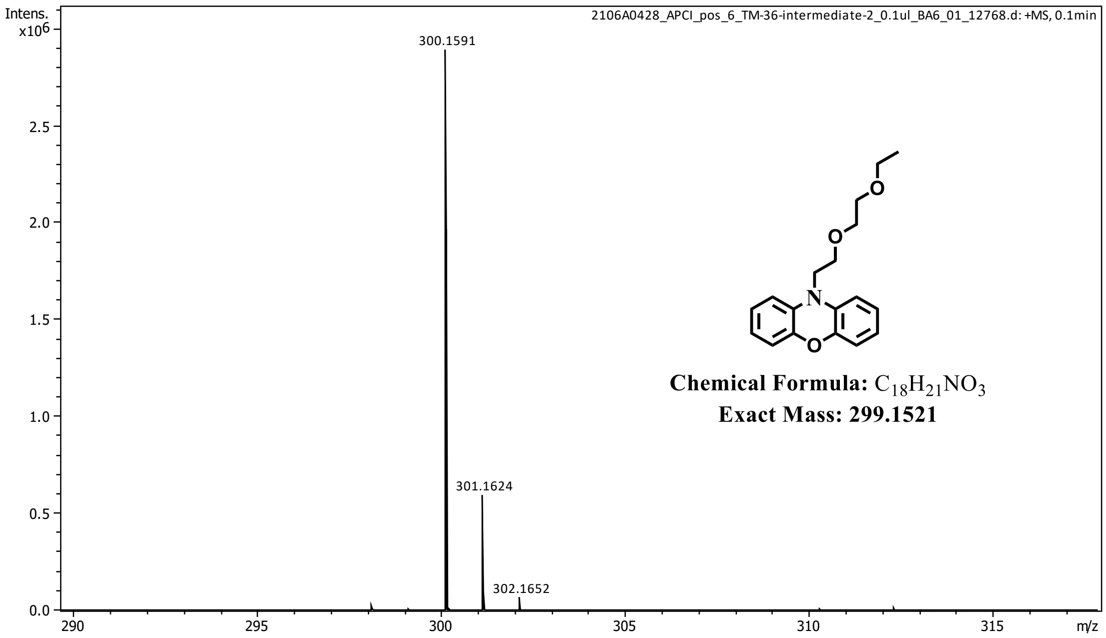

Intens.
x10^6
2106A0428_APCI_pos_6_TM-36-intermediate-2_0.1ul_BA6_01_12768.d: +MS, 0.1min
300.1591
301.1624
302.1652
m/z
Chemical Formula: C18H21NO3
Exact Mass: 299.1521

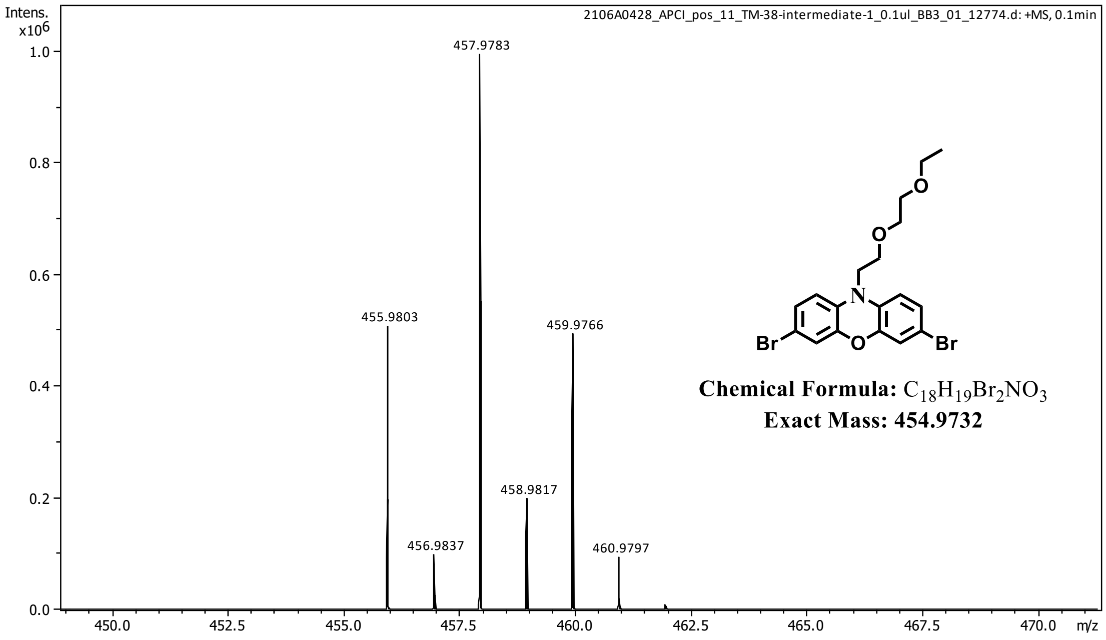

Intens.
x10^6
2106A0428_APCI_pos_11_TM-38-intermediate-1_0.1ul_BB3_01_12774.d: +MS, 0.1min
457.9783
455.9803
459.9766
458.9817
456.9837
460.9797
m/z
Br
Br
Chemical Formula: C18H19Br2NO3
Exact Mass: 454.9732

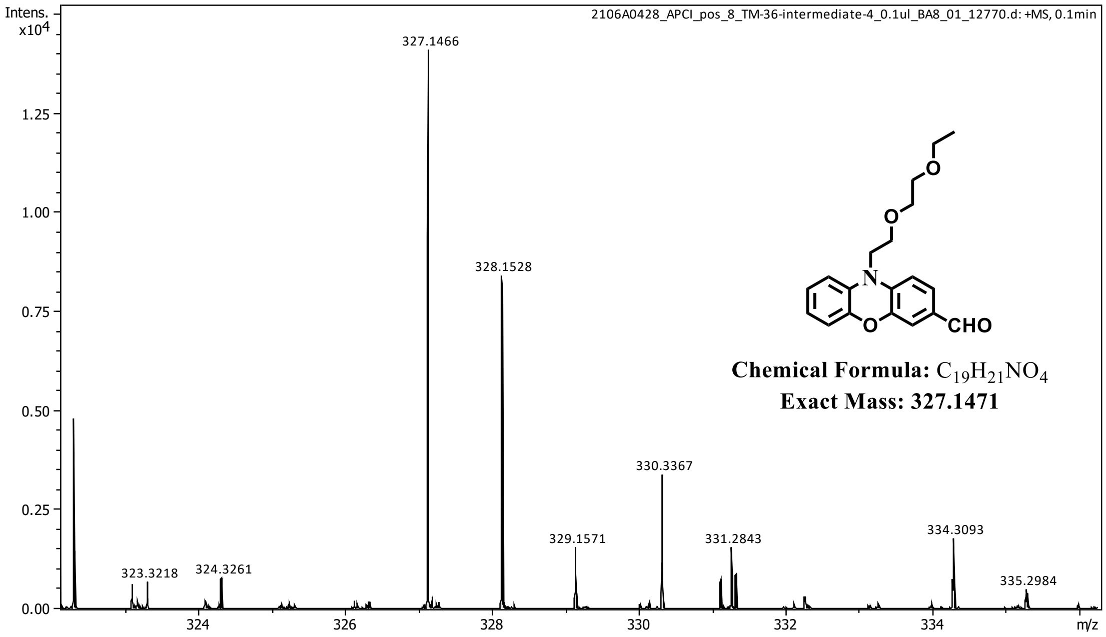
Intens.
x10^4
2106A0428_APCI_pos_8_TM-36-intermediate-4_0.1ul_BA8_01_12770.d: +MS, 0.1min
327.1466
328.1528
330.3367
329.1571
331.2843
334.3093
323.3218
324.3261
335.2984
1.25
1.00
0.75
0.50
0.25
0.00
324
326
328
330
332
334
m/z
N
O
O
O
CHO
Chemical Formula: $C_{19}H_{21}NO_4$
Exact Mass: 327.1471

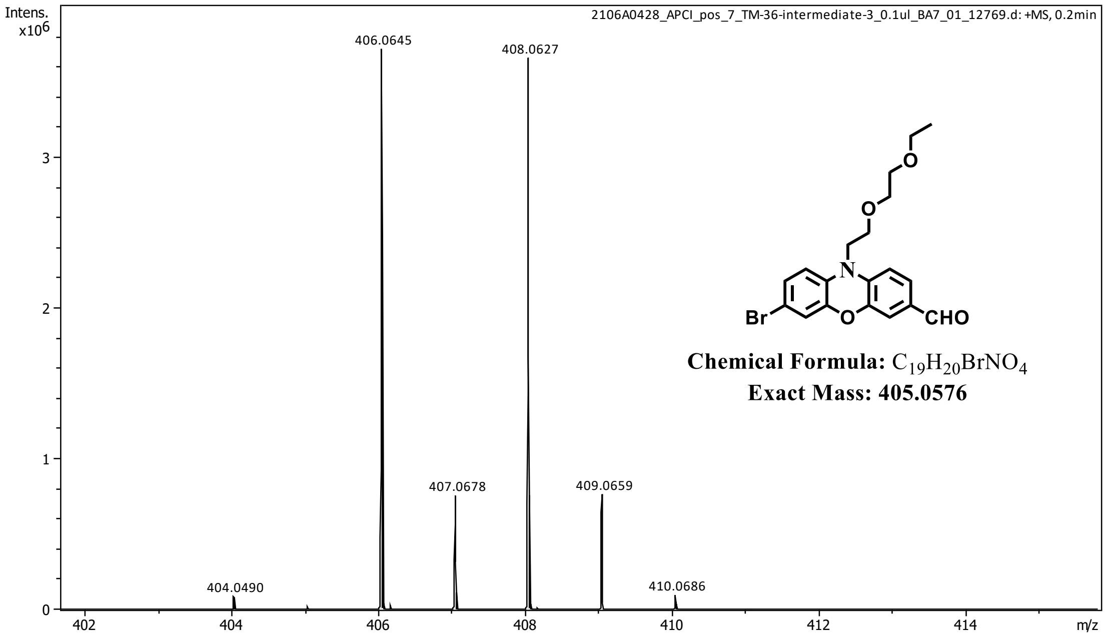
Intens.
x10^6
2106A0428_APCI_pos_7_TM-36-intermediate-3_0.1ul_BA7_01_12769.d: +MS, 0.2min
406.0645
408.0627
407.0678
409.0659
404.0490
410.0686
3
2
1
0
402
404
406
408
410
412
414
m/z
N
O
O
O
Br
CHO
Chemical Formula: $C_{19}H_{20}BrNO_4$
Exact Mass: 405.0576

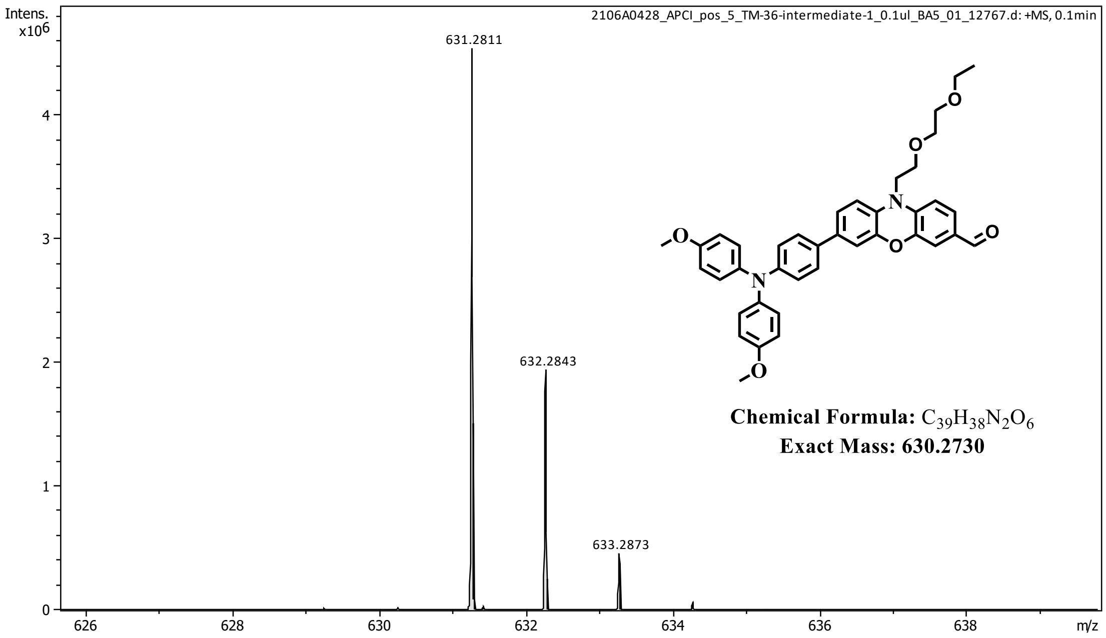

Intens.
x10^6
2106A0428_APCI_pos_5_TM-36-intermediate-1_0.1ul_BA5_01_12767.d: +MS, 0.1min
631.2811
632.2843
633.2873
m/z
Chemical Formula: C39H38N2O6
Exact Mass: 630.2730

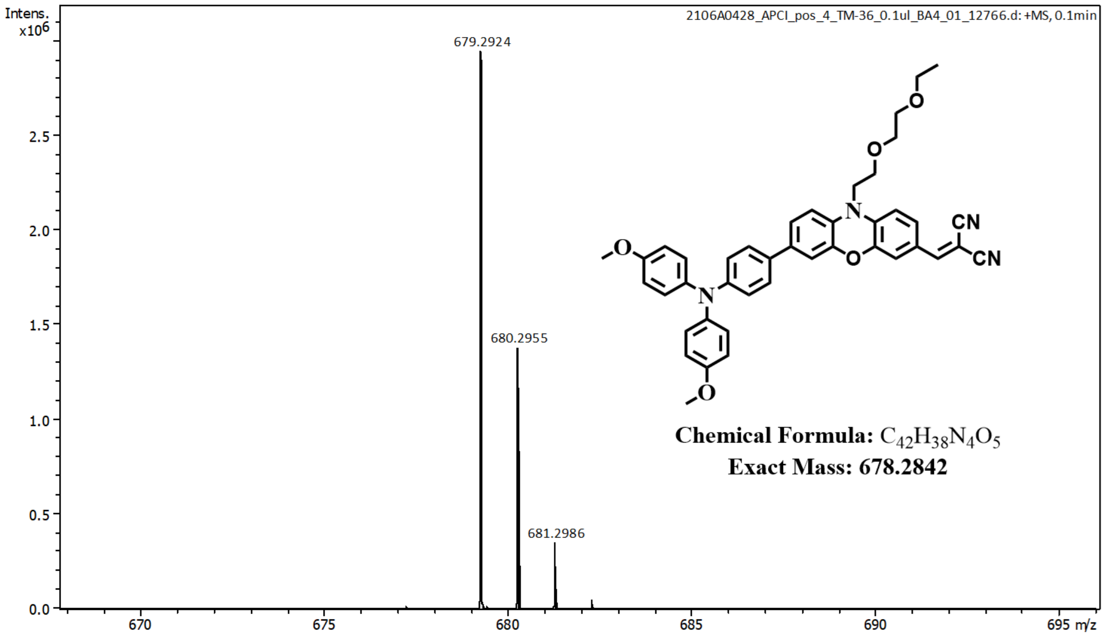

Intens.
x10^6
2106A0428_APCI_pos_4_TM-36_0.1ul_BA4_01_12766.d: +MS, 0.1min
679.2924
680.2955
681.2986
m/z
CN
CN
Chemical Formula: C42H38N4O5
Exact Mass: 678.2842

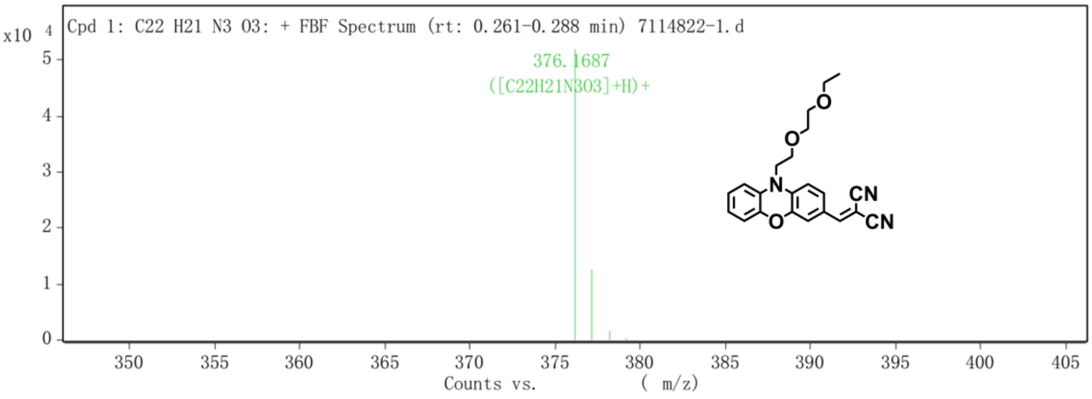

x10 4
Cpd 1: C22 H21 N3 O3: + FBF Spectrum (rt: 0.261-0.288 min) 7114822-1.d
376.1687
([C22H21N3O3]+H)+
CN
CN
Counts vs. (m/z)

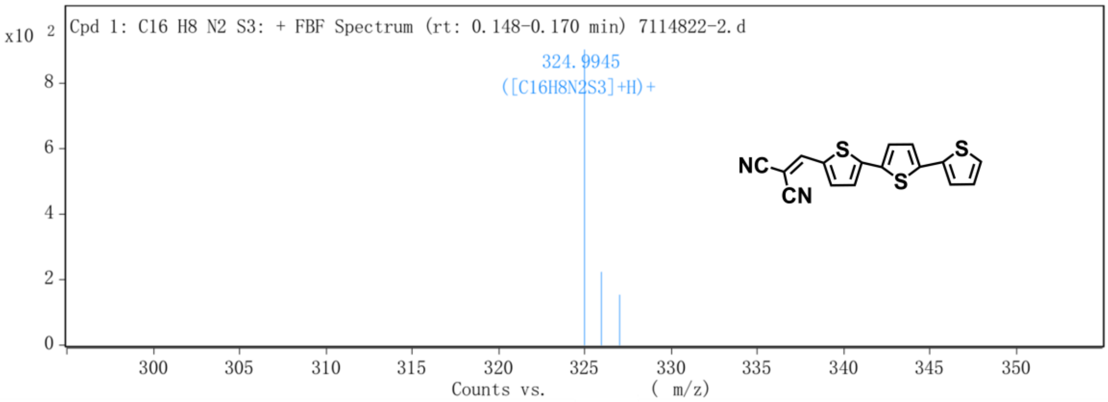
x10 2
Cpd 1: C16 H8 N2 S3: + FBF Spectrum (rt: 0.148-0.170 min) 7114822-2.d
324.9945
([C16H8N2S3]+H)+
NC
CN
S
S
S
Counts vs. (m/z)

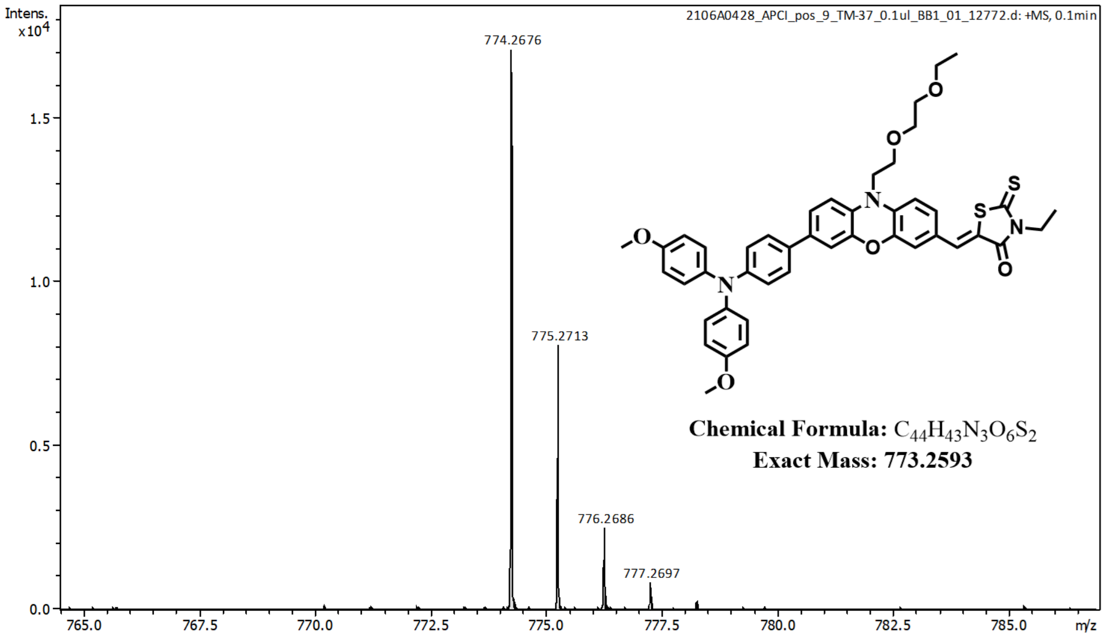
Intens.
x10^4
2106A0428_APCI_pos_9_TM-37_0.1ul_BB1_01_12772.d: +MS, 0.1min
774.2676
775.2713
776.2686
777.2697
m/z
Chemical Formula: C44H43N3O6S2
Exact Mass: 773.2593
