# Supplementary material for: Constructing molecular bridge for high-efficiency and stable perovskite solar cells based on P3HT
Source: Nat Commun. 2022 Nov 17;13:7020. doi: 10.1038/s41467-022-34768-7 (PMC9672063; doi:10.1038/s41467-022-34768-7)
Supplement: Supplementary file 3 — Solar Cells Reporting Summary [file 41467_2022_34768_MOESM3_ESM.pdf]

Corresponding author(s): *Double-blind peer review submissions: write DBPR and your manuscript number here instead of author names.*

Solar Cells Reporting Summary

Nature Research wishes to improve the reproducibility of the work that we publish. This form is intended for publication with all accepted papers reporting the characterization of photovoltaic devices and provides structure for consistency and transparency in reporting. Some list items might not apply to an individual manuscript, but all fields must be completed for clarity.

For further information on Nature Research policies, including our data availability policy, see Authors & Referees.

Experimental design

Please check: are the following details reported in the manuscript?

1. Dimensions

Item	Yes	No	Response
Area of the tested solar cells	☒	☐	The device area is 0.125 cm2
Method used to determine the device area	☒	☐	Metal mask of 0.125 cm2 is used during metal electrode fabrication.

2. Current-voltage characterization

Item	Yes	No	Response
Current density-voltage (J-V) plots in both forward and backward direction	☒	☐	The plots are provided in the manuscript.
Voltage scan conditions<br>*For instance: scan direction, speed, dwell times*	☐	☒	The scanning speed is 100 mV/s and the bias ranges is from −0.2 V to 1.2 V under reverse and forward voltage scan.
Test environment<br>*For instance: characterization temperature, in air or in glove box*	☒	☐	All J–V results are measured in a nitrogen-filled glovebox.
Protocol for preconditioning of the device before its characterization	☐	☒	No preconditioning is used in this work.
Stability of the J-V characteristic<br>*Verified with time evolution of the maximum power point or with the photocurrent at maximum power point; see ref. 7 for details.*	☒	☐	Stabilized PCEs of solar cells are provided.

3. Hysteresis or any other unusual behaviour

Item	Yes	No	Response
Description of the unusual behaviour observed during the characterization	☒	☐	Very minor (2.6%) hysteresis is observed for devices.
Related experimental data	☒	☐	J-V curves under reverse and forward scans are provided.

4. Efficiency

Item	Yes	No	Response
External quantum efficiency (EQE) or incident photons to current efficiency (IPCE)	☒	☐	EQE curves are provided.
A comparison between the integrated response under the standard reference spectrum and the response measure under the simulator	☒	☐	The staedy-state PCE values from EQE are compared to PCE values from J-V measurements, with ~ 0.6% difference.
For tandem solar cells, the bias illumination and bias voltage used for each subcell	☐	☒	Tandem solar cells are not covered in this paper.

5. Calibration

Item	Yes	No	Response
Light source and reference cell or sensor used for the characterization	☒	☐	The J–V characteristics of the devices were measured with a Keithley 2440 source under a simulated AM1.5G spectrum. With a solar simulator (Newport, 91160), the light intensity was calibrated using a standard silicon solar cell device by the NREL.
Confirmation that the reference cell was calibrated and certified	☒	☐	The reference cells are calibrated and certified.

Calculation of spectral mismatch between the reference cell and the devices under test

☐ Yes
☒ No

The light spectrum used for measurements matches well with the reference silicon cell, we do not calculated the spectral mismatch between the reference cell and the tested cells.

6. Mask/aperture

Size of the mask/aperture used during testing

☒ Yes
☐ No

Metal aperture mask with area of 0.07 cm2 is used for testing.

Variation of the measured short-circuit current density with the mask/aperture area

☐ Yes
☒ No

All J-V curves are measured with mask.

7. Performance certification

Identity of the independent certification laboratory that confirmed the photovoltaic performance

☐ Yes
☒ No

Solar cell efficiency is not certified.

A copy of any certificate(s)
Provide in Supplementary Information

☐ Yes
☒ No

No certificate(s).

8. Statistics

Number of solar cells tested

☒ Yes
☐ No

This have been stated in the manuscript.

Statistical analysis of the device performance

☒ Yes
☐ No

This have been stated in the manuscript.

9. Long-term stability analysis

Type of analysis, bias conditions and environmental conditions
For instance: illumination type, temperature, atmosphere humidity, encapsulation method, preconditioning temperature

☒ Yes
☐ No

This have been stated in the manuscript.
